# Unique CLR expression patterns on circulating and tumor-infiltrating DC subsets correlated with clinical outcome in melanoma patients

**DOI:** 10.3389/fimmu.2022.1040600

**Published:** 2022-10-24

**Authors:** Eleonora Sosa Cuevas, Jenny Valladeau-Guilemond, Stephane Mouret, Benoît Roubinet, Florence de Fraipont, Ludovic Landemarre, Julie Charles, Nathalie Bendriss-Vermare, Laurence Chaperot, Caroline Aspord

**Affiliations:** ^1^ Etablissement Français du Sang Auvergne-Rhône-Alpes, R&D Laboratory, Grenoble, France; ^2^ Institute for Advanced Biosciences, Team: Epigenetics, Immunity, Metabolism, Cell Signaling & Cancer, Inserm U 1209, CNRS UMR 5309, Université Grenoble Alpes, Grenoble, France; ^3^ Univ Lyon, Université Claude Bernard Lyon 1, INSERM 1052, CNRS 5286, Centre Léon Bérard, Centre de Recherche en Cancérologie de Lyon, Lyon, France; ^4^ Dermatology, Allergology & Photobiology Department, CHU Grenoble Alpes, Grenoble, France; ^5^ GLYcoDiag, Orléans, France; ^6^ Medical Unit of Molecular genetic (Hereditary Diseases and Oncology), Grenoble University Hospital, Grenoble, France

**Keywords:** CLR, human DC subsets, melanoma, immune subversion, glycan, cDC1s, cDC2s, pDCs

## Abstract

Subversion of immunity by tumors is a crucial step for their development. Dendritic cells (DCs) are strategic immune cells that orchestrate anti-tumor immune responses but display altered functions in cancer. The bases for such DCs’ hijacking are not fully understood. Tumor cells harbor unusual glycosylation patterns of surface glycoproteins and glycolipids. DCs express glycan-binding receptors, named C-type lectin receptors (CLR), allowing them to sense changes in glycan signature of their environment, and subsequently trigger a response. Recognition of tumor glycans by CLRs is crucial for DCs to shape antitumor immunity, and decisive in the orientation of the response. Yet the status of the CLR machinery on DCs in cancer, especially melanoma, remained largely unknown. We explored CLR expression patterns on circulating and tumor-infiltrating cDC1s, cDC2s, and pDCs of melanoma patients, assessed their clinical relevance, and further depicted the correlations between CLR expression profiles and DCs’ features. For the first time, we highlighted that the CLR repertoire of circulating and tumor-infiltrating cDC1s, cDC2s, and pDCs was strongly perturbed in melanoma patients, with modulation of DCIR, CLEC-12α and NKp44 on circulating DCs, and perturbation of Dectin-1, CD206, DEC205, DC-SIGN and CLEC-9α on tumor-infiltrating DCs. Furthermore, melanoma tumor cells directly altered CLR expression profiles of healthy DC subsets, and this was associated with specific glycan patterns (Man, Fuc, GlcNAc) that may interact with DCs through CLR molecules. Notably, specific CLR expression profiles on DC subsets correlated with unique DCs’ activation status and functionality and were associated with clinical outcome of melanoma patients. Higher proportions of DCIR-, DEC205-, CLEC-12α-expressing cDCs were linked with a better survival, whereas elevated proportions of CD206-, Dectin1-expressing cDCs and NKp44-expressing pDCs were associated with a poor outcome. Thus, melanoma tumor may shape DCs’ features by exploiting the plasticity of the CLR machinery. Our study revealed that melanoma manipulates CLR pathways to hijack DC subsets and escape from immune control. It further paved the way to exploit glycan-lectin interactions for the design of innovative therapeutic strategies, which exploit DCs’ potentialities while avoiding hijacking by tumor, to properly reshape anti-tumor immunity by manipulating the CLR machinery.

## Introduction

Interactions between tumor and immune cells are complex and dynamic processes. Avoiding immune destruction is a hallmark of cancer and a crucial step for its development (1). Melanoma has the highest potential among cancers to induce specific anti-tumor responses because of its high mutational rate. Yet, even though the survival of melanoma patients has greatly improved with targeted therapies and immune checkpoint blockade (ICB) (2–4), long-term control of the tumor remains a challenge. Interestingly, distinct immune cell populations in blood or accumulated at the tumor site (T cells, DC subsets, regulatory T cells, γδT cells) differentially influence patient’s clinical outcome (5–8) and correlate with risk of relapse and/or response to immunotherapies (9, 10). These discoveries emphasize the antagonistic anti- or pro-tumoral roles of different immune cell populations and their importance for short- and long-term tumor control in melanoma.

Among immune cells enrolled in the tumor microenvironment, dendritic cells (DCs) are strategic antigen-presenting cells (APCs) that connect innate and adaptive immunity. Given their unique ability to uptake antigens, perform cross-presentation, and activate antigen-specific adaptive immunity, DCs trigger and shape subsequent anti-tumor immune responses (11, 12). There are three main DC subsets in human peripheral blood and lymphoid tissues: conventional DCs type 1 (CD141/BDCA3^+^ cDC1s) and 2 (CD1c/BDCA1^+^ cDC2s), and plasmacytoid DCs (CD303/BDCA2^+^ pDCs) (11, 13). They differ in surface markers expression, localization, cross-presentation capacity, and cytokines secretion (14) allowing them to specifically induce suitable immune responses. cDC1s are the main producers of type III IFN after Toll-like receptor (TLR)-3 signaling (15, 16), possess a high cross-presentation capacity through Clec-9α (17) and induce efficient CD8^+^ cytotoxic T cell responses (17, 18). cDC2s are specialized in the production of IL-12p70 after TLR4 or TLR8 stimulation and induce CD4^+^ T cell responses (19). pDCs are the major producers of type I IFN after TLR7 or TLR9 stimulation and harbor pleiotropic immunomodulatory functions (20, 21). DCs harbor a crucial role in launching anti-tumor responses, especially in melanoma. The density of mature DCs in primary cutaneous melanoma is a strong independent prognostic factor (22). The abundance of tumor-infiltrating cDC2s can be an indicator for protective CD4^+^ T cell quality and better ICB response (23). pDCs can directly attack tumor cells in a TNF-related apoptosis-inducing ligand (TRAIL)-dependent manner (24, 25) or through activation of anti-tumor T-cells (25, 26). cDC1s are essential for initiation and maintenance of anti-tumor responses but also for efficacy of cancer therapies (16, 27, 28). However, tumors can take advantage of DCs’ versatility through suppressive pathways and subvert subsequent anti-tumor responses (10, 29, 30). In melanoma, the three DC subsets infiltrate the tumor site (7). While cDC1s were reported to be functional and linked with a favorable outcome, cDC2s and pDCs display altered features (30, 31), trigger pro-tumor regulatory and Th2 immune responses (5), impact DC cross-talk with anti-tumor immune effector cells (NK, T cells and γδ T cells) (5, 6, 32), and have been associated with poor clinical outcomes (5, 7, 27, 33). However, bases for such DCs’ hijacking in melanoma remain elusive.

The immune response’s orientation depends on DCs’ sensing of their environment. Indeed, DCs recognize pathogen- but also damage-associated molecular patterns (PAMPs and DAMPs respectively) through specific pattern recognition receptors (PRRs) such as TLRs, nucleotide-binding oligomerization domains-like receptors (NLRs), retinoic acid-inducible gene-I like receptors (RLRs), cytosolic DNA sensors (CDSs) and C-type lectin receptors (CLRs) (13). Each DC subset expresses specific PRRs that give them complementary yet specific functions after stimulation (34). It is well documented that glycans on glycoproteins and glycolipids are altered in tumor cells (35), and it turns out that immune cells (especially DCs) express glycan-binding receptors, lectins among which C-type lectin receptors (CLR) (36–38). Thus, DCs can sense changes or abnormalities of glycan signature in their environment, and subsequently trigger a response. CLRs bind to such carbohydrate structures (glycans) exposed at the surface of self or non-self-cells through carbohydrate-recognition domains (CRDs) predominantly in a calcium-dependent manner (37, 39). CLRs can also recognize various forms of ligands independently of their glycosylation status, such as DAMPs released by necrotic cells. Indeed, CLEC-9α can recognize F-actin (40) and MINCLE binds SAP-130 (41). Recognition of self-ligands favors homeostasis and tolerance, whereas non-self-ligands usually induce immunity and inflammation (39, 42). Depending on intra-cellular signaling domains, CLRs are separated into different subgroups (37, 43, 44). Immunoreceptor tyrosine-based activation motif (ITAM)-coupled CLRs (Dectin-2/CLEC6A), and hemi-ITAM (hemITAM)-bearing CLRs (Dectin1/CD369/CLEC7A, DNGR1/CD370/CLEC9A) trigger activation kinases such as SYK, whereas immunoreceptor tyrosine-based inhibitory motif (ITIM)-bearing CLRs (DCIR/CD367/CLEC4A, CD371/CLEC12A) act by recruiting tyrosine phosphatases SHP-1/2. The remaining CLRs (MMR/CD206, DEC205/CD205, Langerin/CD207, BDCA2/CD303/CLEC4C) recruit adaptors bearing ITAM motifs such as FcγRIIα/CD32 or lack typical signaling motifs (DC-SIGN/CD209) and harbor major roles in endocytosis, antigen processing and presentation to T cells (37, 43). After glycan uptake by DCs, CLRs are involved in the induction of specific signaling pathways, either by activating nuclear factor κB (NF-κB) (frequently through CLRs coupled to or bearing ITAM or hemITAM motifs) (43) or by modulating pre-existing TLR signaling (through ITIM-bearing CLRs) (45, 46). Activation of CLR signaling modulates antigen uptake, co-stimulatory molecules expression and cytokine production by DCs, thereby fine-tuning adaptive immune response (34, 43, 44). Importantly, a same CLR can have dual outcome on immune modulation, such as DNGR-1 that can act *via* ITAM but can recruit SHP1 in some context, leading to immunity or inflammation (47). CLRs are pivotal in the shaping of immune responses, translating a variety of glycan structures into a variety of effects ranging from immune suppression to potent immune activation (38). Thus, recognition of tumor glycans by CLRs on DCs could mediate antigen internalization, processing and presentation leading to induction of anti-tumor responses (48), but also trigger immune evasion. Indeed, upon TLR7/9 stimulation, BDCA2 (49) expressed by pDCs forms a complex with ITAM-bearing adaptor FcϵRIγ and down-regulates IFNα production through BCR-like signaling cascades (50). Additionally, other receptors uniquely expressed by human pDCs such as ILT7/CD85g (51) and NKp44/CD336 (52) can also dampen IFNα production by pDCs which leads to regulatory T cell expansion and could favor tumor immune escape (5, 53). Non-self or transformed-self ligands (present on pathogens or tumor cells) can also mimic self-inhibitory signals to favor escape from immune surveillance (35, 37, 54, 55). A study revealed the key role of Dectin1/Gal9 axis on promoting immune tolerance in pancreatic carcinoma (55). Dectin-1 can ligate the lectin galectin 9 in pancreatic ductal adenocarcinoma (PDA), which results in tolerogenic macrophage programming and adaptive immune suppression. Disruption of the Dectin 1-galectin 9 axis reprogrammed efficient T-cell mediated anti-tumor immunity. Melanoma tumor cells exhibit many CLR ligands and display aberrant glycosylation patterns (56–58), yet the impact of such tumor glyco-code on the CLR machinery of DCs remains unexplored.

As tumor cells exhibit aberrant glycan signatures (35) and CLRs are crucial for DCs to shape and polarize immune responses (36, 37), we wondered if abnormal glycosylation of tumor surface glycoproteins and glycolipids could modulate DC activity through CLR signaling and subsequently subvert anti-tumor immune responses in the context of melanoma. Here we investigated CLR’s expression patterns on DC subsets from melanoma patient’s blood and tumor infiltrate, and assessed their clinical relevance. We further explored correlations between CLR expression profiles on DCs and DCs’ features (basal activation status) and functionality upon TLR triggering. Our study reveals that melanoma tumors may exploit CLR pathways to hijack DC subsets and escape from immune control. It further paves the way to exploit the CLR/glycan axis in the context of melanoma to design new therapeutic strategies exploiting DCs’ potentialities while avoiding hijacking by the tumor, to restore potent anti-tumor immunity and improve patient’s clinical outcome.

## Material and methods

### Melanoma patients and control’ samples

This protocol was conformed to the French Blood Service’s (EFS-AuRA) Institutional Review Board and the ethics committee of Grenoble University Hospital (CHU-Grenoble) and declared under the reference #DC-2008-787. Written informed consent was acquired from all participants prior to their participation in this study. Blood samples were obtained from stage I-IV melanoma patients (n=26) and healthy donors (HD, n=77). Peripheral blood mononuclear cells (PBMCs) were isolated using Ficoll-Hypaque density gradient centrifugation (Eurobio). Lymph node or cutaneous metastatic tumors were obtained from 22 melanoma patients (naïve of treatment by immunotherapies). Tonsils obtained from patients that underwent tonsillectomy (n=9) were used as a tissue control. Tumor samples and tonsils were reduced to cell suspensions by enzymatic digestion with 2 mg.ml^-1^ collagenase-D (Roche) 20 U.ml^-1^ DNase (Sigma) and mechanical disruption. The resulting cell suspensions were filtered and washed. Blood and tissue samples were biobanked and stored in liquid nitrogen at -196˚C. Clinical features of melanoma patients are stated in **Supplementary Table 1** (blood samples) and **Supplementary Table 2** (tumor samples). For the HD cohort, the mean age was 43 years, median age 46 years, with min = 18 and max = 68 years. For the patient blood cohort, the mean age was 56 years, median age 49 years, with min = 35 - max = 89 years. For the patient tumor cohort, the mean age was 55 years, median age 59 years, with min = 25 - max = 80 years.

### Co-culture of tumor cells or tumor-derived supernatants with purified DC subsets derived from healthy donors

SK-MEL-5, SK-MEL-28, SK-MEL-24, A375, COLO829, A7, Malme-3M, CHL-1, HT-144 and Hs940.T melanoma cell lines were purchased from American Type Culture Collection (ATCC). Cell cultures were grown in RPMI 1640 GLUTAMAX I supplemented with gentamicin (20µg/mL), non-essential amino acids (MEM 1X) (Invitrogen), sodium pyruvate (1mM) (Sigma) and 10% heat-inactivated FCS in a humidified incubator maintained at 37°C with 5% CO_2_ atmosphere. The medium was changed every other day and the cells were cultured until 70-80% confluence when they were used in the experiments. Tumor supernatants were collected, having changed the medium 24h prior, and stored at -80°C. Tumor cells were checked for *Mycoplasma* contamination using the MycoAlert PLUS detection kit (Lonza). PanDCs (containing a mix of cDC2s, cDC1s and pDCs) were purified from frozen PBMCs derived from healthy donors using the EasySep™ human panDCs pre-enrichment kit (StemCell). PanDCs represented more than 70% of Lin^-^HLA-DR^+^ cells analyzed by flow cytometry. For co-culture experiments, tumor cells were trypsinated using Trypsin/EDTA (StemCell), washed, and seeded in 48-well flat bottom plates. Purified human panDCs were co-cultured with or without confluent tumor cells or tumor-derived supernatants at 1.10^6^/ml in 48-well flat bottom plates for 2 or 20 hours at 37°C with 5% CO_2_ followed by cell staining to assess CLR expression by DC subsets using flow cytometry. We used a tumor/DC ratio of 1:10 to 1:4, as we putted 500.000 PanDCs on a confluent layer of 50.000 to 125.000 tumor cells depending on the cell line, and the tumor-derived supernatants represented 50% of the total medium of the DCs.

### Flow cytometry analysis of CLR expression on human DCs

CLR expression was assessed on thawed controls’ or melanoma patients’ samples (PBMCs, cell suspensions from tonsils, tumor-infiltrating immune cells) and on purified panDCs (derived from co-culture experiments) following staining in PBS 2% fetal calf serum (FCS) and flow cytometry analysis. The combination of fluorochrome-labeled anti-human CD45, Lineage cocktail (CD3/CD14/CD16/CD19/CD20/CD56) (BD or Biolegend), CD11c, HLA-DR antibodies allowed the definition of DC subsets. In addition, BDCA1/CD1c (Beckman), BDCA3/CD141 and BDCA4/CD304 (Miltenyi Biotec) antibodies were used to depict cDC2s, cDC1s and pDCs respectively. Thus, DC populations were identified as alived singlet CD45^+^HLA-DR^+^Lin^-^ cells and subdivided as CD11c^+^BDCA1^+^ cDC2s, CD11c^+^BDCA3^+^ cDC1s and CD11c^-^BDCA4^+^ pDCs. Furthermore, CLR expression on DC subsets was studied using fluorochrome-labeled anti-human DNGR1/CD370/Clec-9α, MMR/CD206, DC-SIGN/CD209 (BD); BDCA2/CD303/Clec-4C (Miltenyi Biotec); Dectin-1/CD369/Clec-7α, CD371/Clec-12α, DEC-205/CD205, Langerin/CD207, FcεRIα (Biolegend); FcɣRIIα/CD32, ILT7/CD85g (Invitrogen); NKp44/CD336 (Beckman Coulter); DCIR/CD367/Clec-4α (R&D systems) antibodies. Later on, stained cells were fixed with FACS lysing solution (BD) and further analyzed using LSRII Flow Cytometer and FACSDiva software v.9 (BD). Isotype controls were used to differentiate positive cells from nonspecific background staining (CD45^+^ cells also served to determine the threshold of positivity). Dead cells were excluded with Live and Dead staining (Invitrogen). Both proportions and Mean Fluorescence Intensities (MFIs) of positive cells were analyzed. MFIs were interpreted only when the percentage of positive cells was ≥ 10%. To ensure quality control during the study, we performed a standardization of the fluorescence intensities using cytometer setup and tracking beads (CST) (BD).

### Assessment of cytokine production by pDCs upon TLR triggering

PBMCs were collected in 96-well U-bottom plates and cultured at 1.10^6^/mL for 5 hours with or without Class-A CpG oligonucleotide ODN-2336 (CpGA, 1µM) (*In vivo*gen). 1 µg.mL-1 of Brefeldin A (BD) was added after 1 hour. Afterwards, cells were stained for surface markers to depict pDCs (CD11c, HLA-DR (BD), Lin, CD45 (Biolegend), BDCA2 (Miltenyi)) and Live and Dead staining (Invitrogen) was used to exclude dead cells. Samples were then fixed and permeabilized according to the manufacturer’s instructions (BD Cytofix/CytopermTM Plus kit) and intracellular cytokine staining was performed using fluorochrome-labeled anti-human antibodies (IFNα (Miltenyi) and IP10 (BioTechne). Analyses were done by flow cytometry using LSRII Flow Cytometer and FACSDIVA software v.9 (BD). Proportions of cytokine-producing pDCs were analyzed.

### GLYcoPROFILE™ of tumor cell lines derived from melanoma patients and healthy melanocytes

The cell suspension resulting from tumor disruption of the patients was put into a culture flask. After 24h, the medium was removed and adherent tumor cells were further cultured. Tumor cells were checked for Mycoplasma contamination using the MycoAlert PLUS detection kit (Lonza) and tumor cell lines with less than ten passages were used for the experiments. The glyco-code of these primary melanoma tumor cells was assessed by performing the GLYcoPROFILE™ with the LEctPROFILE^®^ plates from GLYcoDiag (Orleans, France). The assessment of interactions of lectins with glycans on cell surfaces were achieved according to GLYcoDiag’s protocol (59, 60). When cells grew up to 80–90% confluence in 75 cm2 culture flask, cells were washed with PBS and harvested with a Trypsin/EDTA solution. After washing and centrifugation, the cells were suspended in PBS and labeled with carboxyfluorescein diacetate succinimidylester (CFDASE, Sigma-Aldrich, St. Louis, MO, USA) in PBS. Next, 100 μL of labeled cells (about 2 × 10^5^ cells) were added in each well of the LEctPROFILE^®^ plates (**Supplementary Table 3**) and incubated 2 h at room temperature under gentle agitation. After washing with PBS, fluorescence intensity was measured using a microplate reader (λex = 485 nm, λem = 530 nm, Fluostar OPTIMA, BMG LABTECH, France). In parallel, a calibration curve was achieved with the labeled cells solution to determine the number of cells stayed in interactions with lectins.

### Correlations of CLR expression with DC functional data

To assess the potential link between CLR expression on DC subsets and DCs’ functionality, we performed non-parametric Spearman correlations between the current data and previously published functional studies on DCs from the same samples (dataset published in Sosa Cuevas et al. Clin Transl Immunol 2020). In addition, the protocols used to assess DC basal activation status and intracellular cytokine production by DC subsets were described previously in Sosa Cuevas et al. CTI 2020.

### Statistical analyses

Statistical analyses were performed using Mann-Whitney non parametric *U*-test with Bonferonni corrections for multiple comparisons, Kruskal-Wallis non parametric test with *post hoc* Dunns’ multiple comparison test, and Spearman correlation using Graph Pad Prism software. Data were shown as means and significance threshold was placed at *p*-value < 0.05. Survival analyses (Cox regression, Kaplan-Meier), correlations, heatmaps and Principal Component Analysis (PCA) were performed using the survival, dplyr, survminer, forestmodel, corrplot, gplots, ggbiplot, RColorBrewer, MissMDA and FactoMineR packages using RStudio software R version 4.1.2.

## Results

### Circulating and tumor-infiltrating cDC2s harbor specific CLR profile in melanoma patients

**Figure 1:**
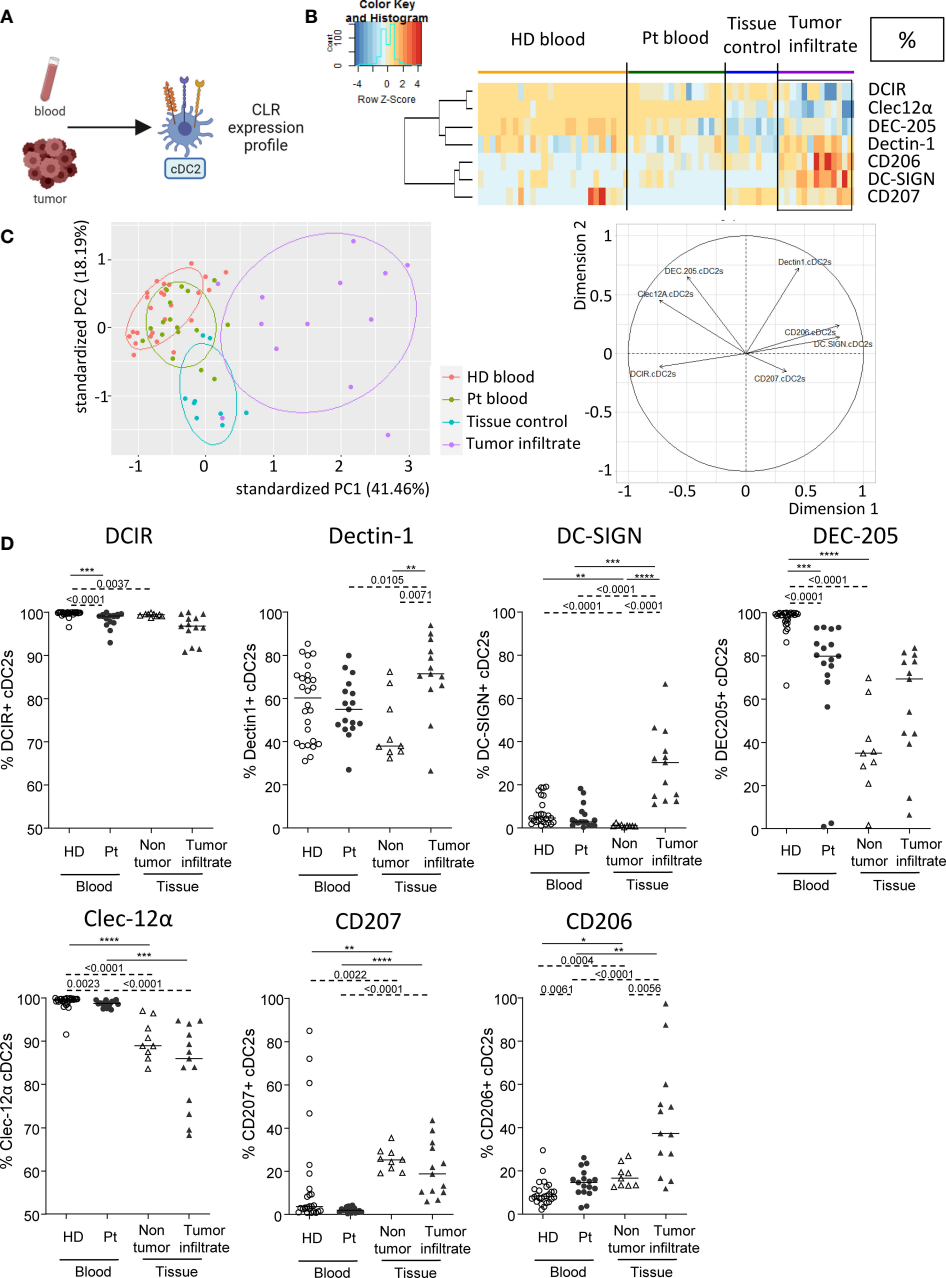
Circulating and tumor-infiltrating cDC2s harbor specific CLR profile in melanoma patients. Circulating and tumor-infiltrating immune cells from melanoma patients and controls were labelled with specific antibodies to depict CD11c^+^BDCA1^+^ cDC2s amongst alive CD45^+^Lin^-^HLA-DR^+^ cells, and their CLR expression was assessed using flow cytometry. **(A)** Schematic illustration of the experiment. CLR expression profile was assessed on cDC2s from blood and tumor samples. **(B)** Heat map based on the percentage of expression of DCIR, Dectin-1, DC-SIGN, DEC-205, Clec-12α, CD207 and CD206 on cDC2s derived from patients’ blood (n=17) and tumors (n=13), and controls’ blood (n=26) and tissue (n=9). **(C)** Principal component analysis (PCA) based on CLR expression on cDC2s of the four groups studied (including graph of variables). **(D)** Expression levels of DCIR, Dectin-1, DC-SIGN, DEC-205, Clec-12α, CD207 and CD206 on cDC2s from the blood of healthy donors (HD, open circles, n=26) and melanoma patients (Pt, filled circles, n=17), and tissue control (open triangles, n=9) and tumor infiltrates of melanoma patients (filled triangles, n=13). Results are expressed as percentages of positive cells within cDC2s. Only significant statistics are shown on the graphs. Bars indicate median. *P*-values were calculated using Mann-Withney (dashed lines) and Kruskal-Wallis (full lines) non parametric tests. * P-value < 0.05 **P-value ≤ 0.01, ***P-value ≤ 0.001, ****P-value ≤ 0.0001.

As CLR are crucial for DCs to sense their environment and subsequently shape immunity, we investigated the basal CLR expression profile on DC subsets in patient’s blood and tumor immune infiltrate by designing a multi-parametric flow cytometry strategy allowing the extensive analysis of specific CLRs of the three major DC subsets (**Supplementary Figure 1**). cDC2s were defined as Lin^-^HLA-DR^+^ CD11c^+^BDCA1^+^ among alive CD45^+^ cells and CLR expression was assessed using flow cytometry (**Figure 1A**). We selected a panel of CLRs known to be expressed on cDC2s: DCIR/CD367/Clec-4α, Dectin-1/CD369/Clec-7α, DC-SIGN/CD209, DEC-205/CD205, Clec-12α/CD371, Langerin/CD207 and MMR/CD206. We first ensure that CLR expression profiles were not disturbed between fresh and frozen samples, allowing combining frozen samples from all groups at the same time with retrospection on clinical outcomes (**Supplementary Figure 2A**). To depict the potential modulation of the CLR profile in the context of melanoma especially in the tumor microenvironment, we compared blood of patients (Pt) with healthy donors (HD), and tumor tissue with control tissue because the CLR profile of DCs in control (HD) conditions differed in blood and tissue. We performed Euclidean distance-based hierarchical clustering (**Figure 1B**; **Supplementary Figure 3A**) and ran PCA analyses (**Figure 1C**; **Supplementary Figure 3B**) to get a global view of CLR profiles in the different groups. Interestingly, heat maps based on basal CLR expression on cDC2s (both percentage and MFI) revealed perturbations of CLR expression mostly on tumor-infiltrating cDC2s when compared to patient’s blood and control tissue (**Figure 1B**; **Supplementary Figure 3A**). Furthermore, tumor-infiltrating cDC2s were placed in a separate area of the PCA analyses (based on PC1 and PC2) when compared to the other three groups (**Figure 1C**; **Supplementary Figure 3B**; left panels). These observations suggest an overall modulation of the CLR profile on tumor-infiltrating cDC2s, mostly driven by levels of Dectin-1, DC-SIGN and CD206 (**Figure 1C**; **Supplementary Figure 3B**; right panels). When going deeper into individual CLR expression, we observed a decrease of the frequency of circulating DEC-205^+^ cDC2s together with a slight diminution of DCIR^+^ cDC2s, whereas slightly higher frequencies of CD206^+^ cDC2s were observed in patients compared to HDs (**Figure 1D**). In addition, circulating cDC2s from patients displayed higher levels of expression (MFI) of Clec-12α when compared to HDs (**Supplementary Figure 3C**). Strikingly, tumor-infiltrating cDC2s exhibited higher levels of Dectin-1, DC-SIGN, DEC-205 or CD206 (in frequencies and/or intensity) when compared to control tissue (**Figure 1D**; **Supplementary Figure 3C**). Observations were not due to age differences, as cohorts were age-matched and CLR profiles in “young” and “elderly” patients were similar (**Supplementary Figure 4**). Thus, the basal CLR expression profile on circulating and tumor-infiltrating cDC2s is drastically modulated in melanoma patients, with a major impact within the tumor microenvironment.

### cDC1s from blood and tumor of melanoma patients display distinct CLR expression profiles compared to controls

**Figure 2:**
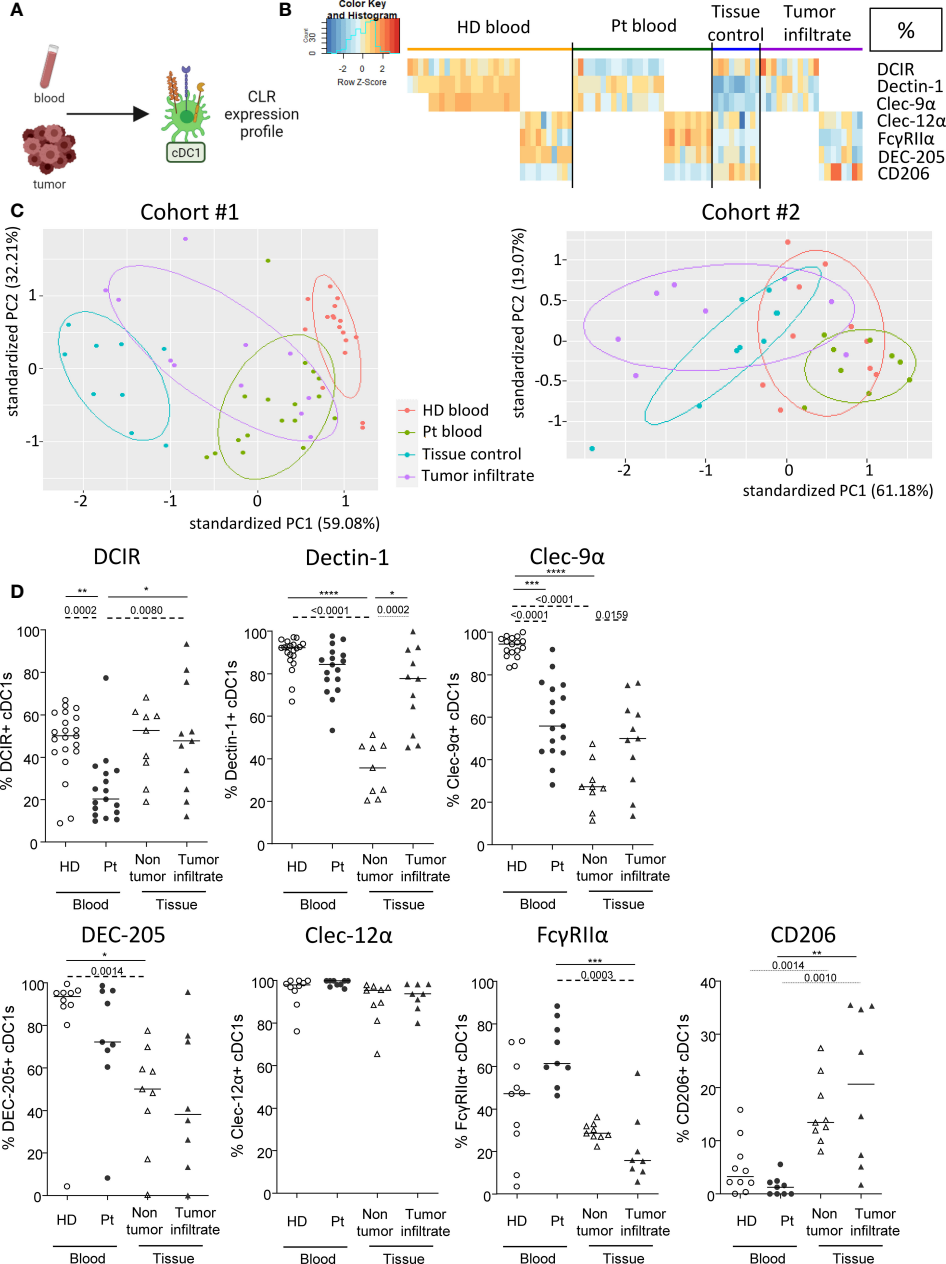
cDC1s from blood and tumor of melanoma patients display distinct CLR expression compared to controls. Circulating and tumor-infiltrating immune cells from melanoma patients and controls were labelled with specific antibodies to depict CD11c^+^BDCA3^+^ cDC1s amongst alive CD45^+^Lin^-^HLA-DR^+^ cells, and their CLR expression was assessed using flow cytometry. **(A)** Schematic illustration of the experiment. CLR expression profile was assessed on cDC1s from blood and tumor. **(B)** Heat map based on the percentage of expression of DCIR, Dectin-1, Clec-9α, DEC-205, Clec-12α, FcγRIIα and CD206 on cDC1s derived from patients’ blood (n=26) and tumors (n=18), and controls’ blood (n=31) and tissue (n=9) (juxtaposition of two non-supervised clustering performed independently on the two sub-cohorts). **(C)** PCA based on CLR expression on cDC1s of the four groups studied for the two distinct data sets (DCIR, Dectin-1, Clec-9α in the left panel; and DEC-205, Clec-12α, FcγRIIα, CD206 in the right panel). **(D)** Expression levels of DCIR, Dectin-1, Clec-9α, DEC-205, Clec-12α, FcγRIIα and CD206 on cDC1s from the blood of healthy donors (HD, open circles, n=31) and melanoma patients (Pt, filled circles, n=26), and tissue control (open triangles, n=9) and tumor infiltrates of melanoma patients (filled triangles, n=26). Results are expressed as percentages of positive cells within cDC1s. Only significant statistics are shown on the graphs. Bars indicate median. *P*-values were calculated using Mann-Withney (dashed lines) and Kruskal-Wallis (full lines) non parametric tests. *P-value ≤ 0.05, **P-value ≤ 0.01, ***P-value ≤ 0.001, **** P-value < 0.0001.

As DC subsets are specialized and complementary in their PRRs’ equipment, we then explored another set of CLRs on cDC1s in patient’s blood and tumor immune infiltrate. cDC1s were depicted as Lin^-^HLA-DR^+^CD11c^+^BDCA3^+^ among alive CD45^+^ cells and CLR expression was measured using flow cytometry (**Figure 2A**). CLR and adaptors studied on cDC1s were the following: DCIR/CD367/Clec-4α, Dectin-1/CD369/Clec-7α, DNGR1/CD370/Clec-9α, DEC-205/CD205, Clec-12α/CD371, FcɣRIIα/CD32 and MMR/CD206. The CLR profiling on cDC1s has been performed on two distinct datasets for each group: DCIR, Dectin-1 and Clec-9α were assessed in one cohort (21 HDs, Patients #1-17 and #27-40; **Supplementary Tables 1** and **2**), and DEC-205, Clec-12α, FcγRIIα and CD206 were analyzed in another cohort (10 HDs, Patients #18-26 and #41-48; **Supplementary Tables 1** and **2**). We performed supervised nonhierarchical clustering (**Figure 2B**, **Supplementary Figure 5A**) and ran PCA analyses for each dataset (**Figure 2C**; **Supplementary Figures 5B, C**). Heat maps based on CLR expression levels on cDC1s revealed distinct patterns of CLR expression on cDC1s in both blood and tumors from patients when compared to control groups (**Figure 2B**; **Supplementary Figure 5A**). Such observation was further sustained by PCA analyses that located circulating and/or tumor-infiltrating cDC1s from patients in distinct areas compared to their respective control group (**Figure 2C**, left panels; **Supplementary Figures 5B, C**). Individual CLR expression further depicted specificities of the CLR profile of cDC1s in patients. In blood, melanoma patients harbored a decrease in frequencies of DCIR^+^ and Clec-9α^+^ cDC1s as well as an increase in expression level (MFI) of Clec-12α on cDC1s when compared to HDs (**Figure 2D**; **Supplementary Figure 5D**). Strikingly, in tumor, higher levels of Dectin-1 and Clec-9α (in both frequencies and intensity) were found on cDC1s in patients when compared to control tissue, even though proportions of Clec-9a remain lower than in HDs (**Figure 2D**; **Supplementary Figure 5D**). Observations were not due to age differences, as cohorts were age-matched and CLR profiles in “young” and “elderly” patients were similar (**Supplementary Figure 4**). Thus, cDC1s from melanoma patients exhibit strong perturbations of the basal CLR expression pattern, both in blood circulation and within tumor microenvironment.

### Circulating pDCs from melanoma patients exhibit modulation of their CLR expression profile compared to controls

**Figure 3:**
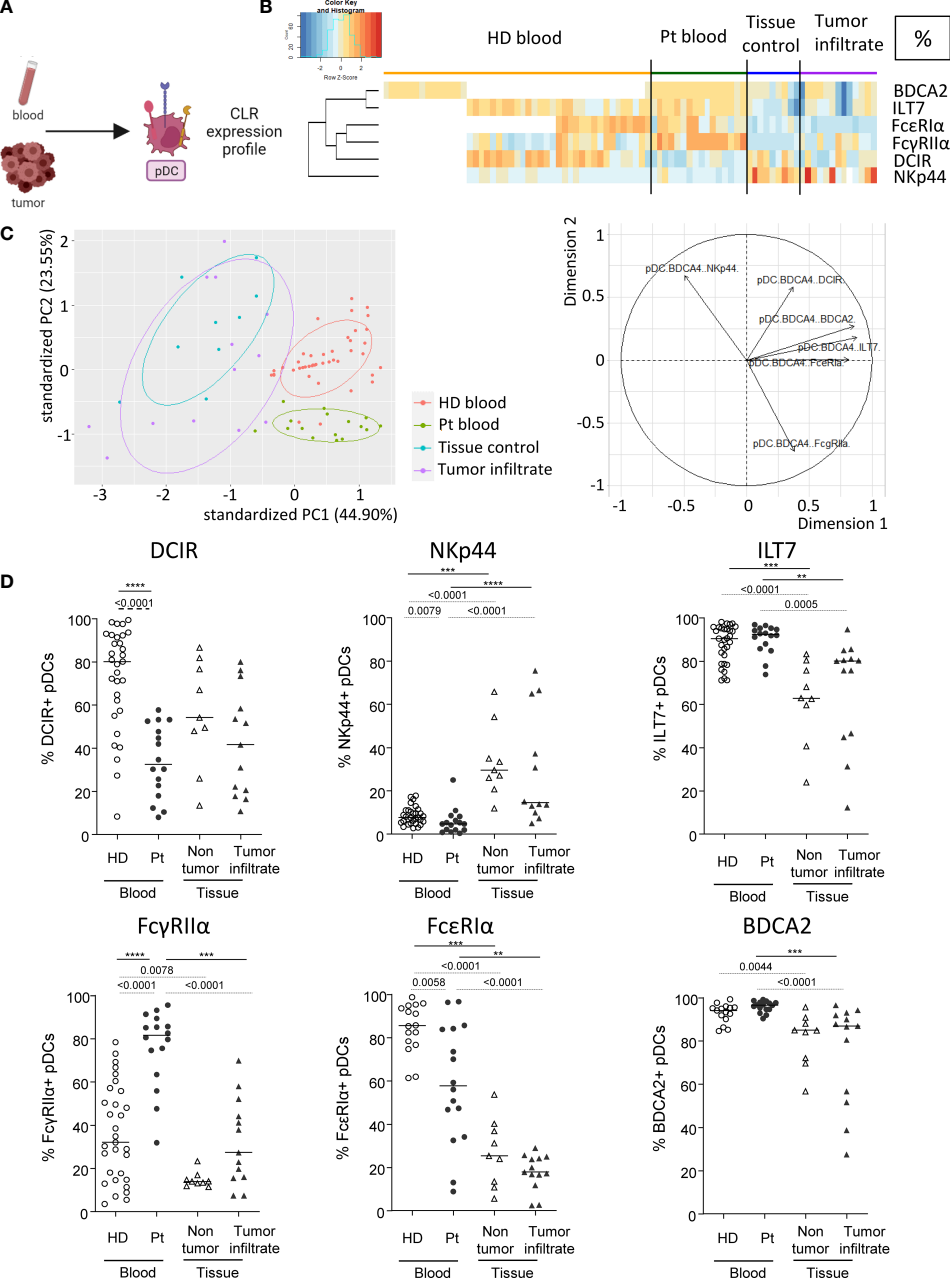
Modulation of CLR expression on circulating and tumor-infiltrating pDCs in melanoma patients. Circulating and tumor-infiltrating immune cells from melanoma patients and controls were labelled with specific antibodies to depict CD11c^-^BDCA4^+^ pDCs amongst alive CD45^+^Lin^-^HLA-DR^+^ cells and their CLR expression was assessed using flow cytometry. **(A)** Schematic illustration of the experiment. CLR expression profile was assessed on pDCs from blood and tumor. **(B)** Heat map based on the percentage of expression of DCIR, NKp44, ILT7, FcγRIIα, FcϵRIα and BDCA2 on pDCs derived from patients’ blood (n=16) and tumors (n=13), and controls’ blood (n=45) and tissue (n=9). **(C)** PCA based on CLR expression on pDCs of the four groups studied (including graph of variables). BDCA2 could not be integrated in this analysis given that it was analyzed in a different dataset from the other CLRs studied for pDCs. **(D)** Expression levels of DCIR, NKp44, ILT7, FcγRIIα, FcϵRIα and BDCA2 on pDCs from the blood of healthy donors (HD, open circles, n=45) and melanoma patients (Pt, filled circles, n=16), and tissue control (open triangles, n=9) and tumor infiltrates of melanoma patients (filled triangles, n=13). Results are expressed as percentages of positive cells within pDCs. Only significant statistics are shown on the graphs. Bars indicate median. *P*-values were calculated using Mann-Withney (dashed lines) and Kruskal-Wallis (full lines) non parametric tests. **P-value ≤ 0.01, ***P-value ≤ 0.001, ****P-value ≤ 0.0001.

We then examined CLR expression on pDCs in patient’s blood and tumor immune infiltrate, as this subset massively infiltrates melanoma tumors and drives poor outcome. pDCs were depicted as Lin^-^HLA-DR^+^CD11c^-^BDCA4^+^ among alive CD45^+^ cells and CLR expression was measured using flow cytometry (**Figure 3A**). CLRs, adaptors and receptors studied on pDCs were DCIR/CD367/Clec-4α, NKp44/CD336, ILT7/CD85g, FcɣRIIα/CD32, FcεRIα and BDCA2/CD303/Clec-4C. Heat maps performed upon Euclidean distance-based hierarchical clustering and based on CLR expression on pDCs showed different patterns of CLR expression in patients, mostly on circulating pDCs when compared to HD blood (**Figure 3B**; **Supplementary Figure 6A**). Moreover, frequencies of CLR-expressing pDCs in patient’s blood were positioned in a separate area of the PCA analysis (based on PC1 and PC2) when compared to HD blood and/or tumor infiltrate (**Figure 3C**; left panel; **Supplementary Figure 6B**), and this distinction seemed to mostly be driven by DCIR and FcɣRIIα expression (**Figure 3C**; right panel). Interestingly, lower frequencies of circulating DCIR^+^, NKp44^+^ and FcεRIα^+^ pDCs were found in patients (**Figure 3D**), whereas an increase of FcɣRIIα (frequency and/or intensity) on circulating pDCs was observed in patients when compared to HDs (**Figure 3D**; **Supplementary Figure 6C**). Surprisingly, no major modulation of the CLR expression profile on tumor-infiltrating pDCs was noticed when compared to control tissues. Nevertheless, we observed in tumors an increase of NKp44 and a decrease of ILT7, FcεRIα and BDCA2 (frequencies and/or intensities) on pDCs when compared to patient’s blood (**Figure 3D**; **Supplementary Figure 6C**). Observations were not due to age differences, as cohorts were age-matched and CLR profiles in “young” and “elderly” patients were similar (**Supplementary Figure 4**). Taken together, these results indicated that the CLR expression profile on circulating pDCs is strongly modulated in melanoma patients.

### DCs subsets derived from HD blood exhibited perturbation of their CLR profile after direct or indirect interactions with melanoma tumor cells

**Figure 4:**
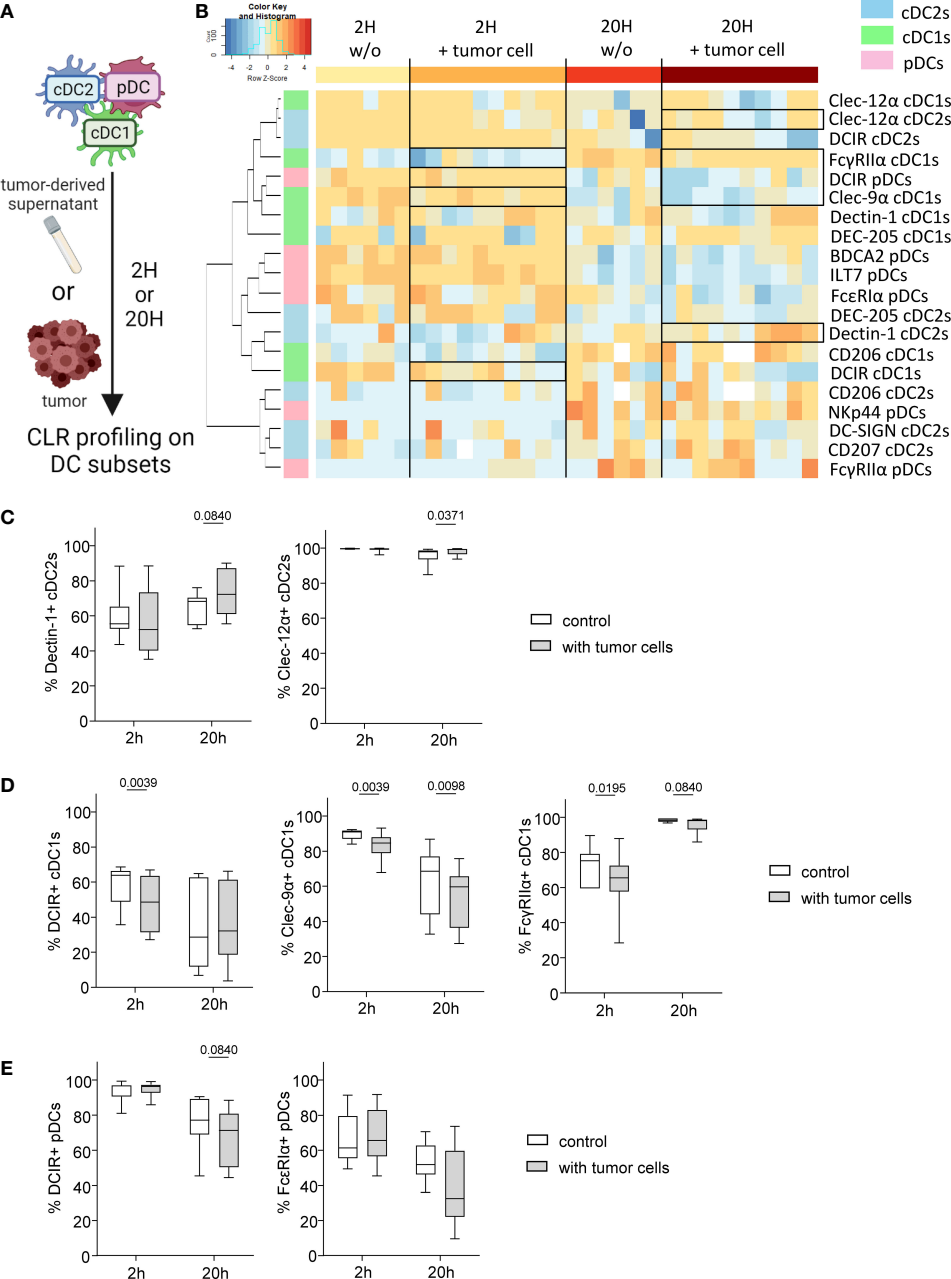
Melanoma tumor cells trigger modulation of CLR profile on « healthy » DC subsets. PanDCs (mixture of the three DC subsets cDC2s, cDC1s, pDCs) were purified from several HD blood and co-cultured with distinct melanoma cell lines (in a tumor/DC ratio of 1:10 to 1:4) or tumor supernatants (50% of total medium) for 2 or 20 hours. CLR expression profile on DC subsets was subsequently assessed using flow cytometry. **(A)** Schematic representation of the experimental layout to investigate the impact of distinct tumor cell lines on CLR expression by healthy panDCs. **(B)** Heat map based on the expression of DCIR, Dectin-1, DC-SIGN, DEC-205, Clec-12α, CD207 and CD206 on cDC2s; DCIR, Dectin-1, Clec-9α, DEC-205, Clec-12α, FcγRIIα and CD206 on cDC1s; and DCIR, NKp44, ILT7, FcγRIIα, FcϵRIα and BDCA2 on pDCs after 2 or 20 hours of culture with (n=10) or without (n=6) distinct tumor cell lines. **(C)** Expression levels of Dectin-1 and Clec-12α on cDC2s purified from HD blood after 2 or 20 hours of co-culture with tumor cell lines (n=8 to 10). **(D)** Expression levels of DCIR, Clec-9α and FcγRIIα on cDC1s purified from HD blood after 2 or 20 hours of co-culture with tumor cell lines (n=10). **(E)** Expression levels of DCIR and FcϵRIα on pDCs purified from HD blood after 2 or 20 hours of co-culture with tumor cell lines (n=8 to 10). **(C-E)** Results are expressed as percentages of positive cells within each DC subset. Only statistics with a *P*-value < 0.1 are shown on the graphs. *P*-values were calculated using Wilcoxon matched-pairs signed rank test.

The previous results prompted us to investigate whether melanoma could directly affect CLR expression on DC subsets. We therefore assessed whether CLR expression on DCs could be modulated by melanoma tumor cells directly through cell-cell interactions or indirectly by tumor secreted factors. We co-cultured *in vitro* purified “healthy” panDCs (mixture of the three DC subsets from HD blood) with tumor cells or tumor-derived supernatants for 2 or 20 hours and subsequently evaluated CLR expression on DC subsets by flow cytometry (**Figure 4A**). We first made sure that DC viability was over 85% (data not shown) and that CLR expression patterns on cDC2s, cDC1s and pDCs within purified PanDCs at T0 were the ones expected based on HDs’ expression (**Supplementary Figure 7A**). Given tumor cell heterogeneity in melanoma, we used ten different tumor cell lines (SK-MEL-5, SK-MEL-28, SK-MEL-24, A375, COLO829, A7, Malme-3M, CHL-1, HT-144 and Hs940.T). We performed Euclidean distance-based hierarchical clustering, and the resulting heat maps illustrated modulations of CLR expression profiles on the three DCs subsets upon contact with melanoma cells (**Figure 4B**) or tumor-derived supernatants (**Supplementary Figure 7B**). Strikingly, we observed that tumor cells or supernatants triggered modulations of CLR expression on DCs (**Figure 4B**; **Supplementary Figure 7B**), occurring with different manner depending on the DC subset. Overall, these modulations were induced by 8-9 out of 10 tumor cell lines, and 6 out of 6 tumor supernatants. Consistently with major perturbations of CLR profile of tumor-infiltrating cDC2s observed in patients, tumor cells rather than supernatants mostly affected cDC2s. Indeed, we observed a very small increase of Clec-12α^+^ cDC2s after 20h of culture with tumor cells (**Figure 4C**), whereas a tendency to higher frequencies of Dectin-1^+^ cDC2s was observed with tumor cells and supernatants when compared to controls (**Figure 4C**; **Supplementary Figure 7C**). Again, consistently with modulation of the CLR profile of cDC1s both in blood and tumor of melanoma patients, cDC1s were affected by both tumor cells and their supernatants. Indeed, lower levels of DCIR^+^, Clec-9α^+^ or FcγRIIα^+^ cDC1s were found after 2 and/or 20h of culture with tumor cells (**Figure 4D**) and/or tumor supernatants (**Supplementary Figure 7D**). Moreover, consistently with previous observations in patients depicting CLR perturbations on pDCs mostly in blood circulation, pDCs were mostly impacted by tumor-derived supernatants, as we observed lower levels of DCIR^+^ and FcϵRIα^+^ pDCs after 20h of culture with tumor supernatants (**Figure 4E**; **Supplementary Figure 7E**). Notably, CLRs modulated on DC subsets by tumor cells/supernatants corroborated changes observed in melanoma patients (**Figures 1**–**3**).

**Figure 5:**
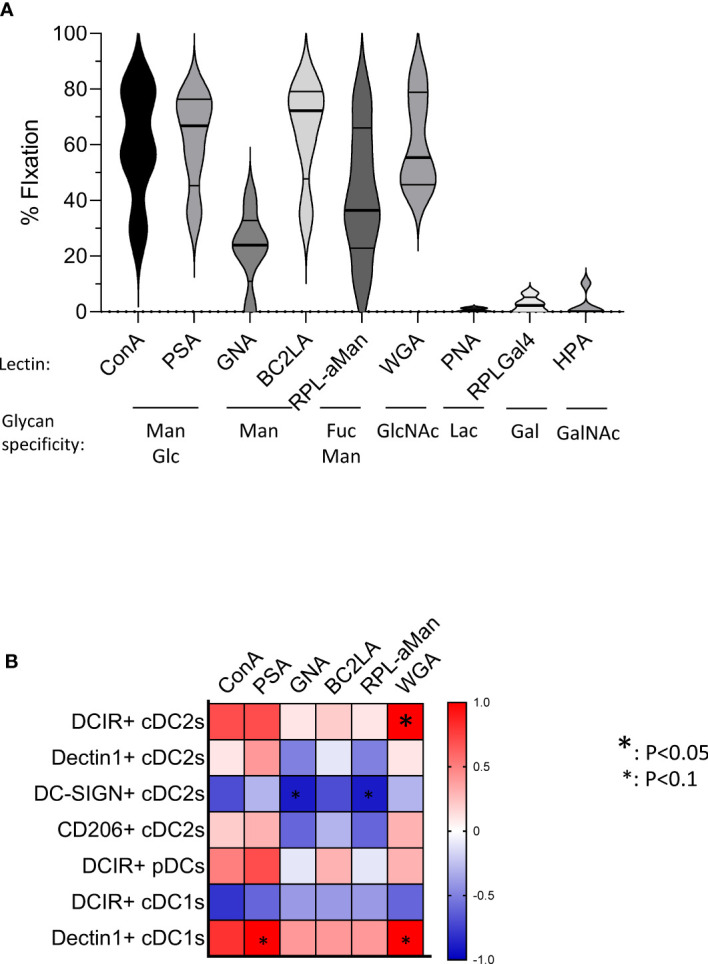
Primary melanoma tumor cells harbor specific glycan profiles that correlate with CLR expression levels on tumor-infiltrating DCs in corresponding patients. GLYcoPROFILE™ (lectin arrays from GLYcoDiag) were performed on primary tumor cell lines derived from melanoma patients. CLR expression profiles were assessed on tumor-infiltrating DC subsets from the corresponding patients by flow cytometry. **(A)** Levels of lectin fixation (indicators of the levels of glycan expression) of ConA, PSA, GNA, BC2LA, RPL-αMan, WGA, PNA, RPL-Gal4 and HPA by tumor cells (n =5) together with their glycan specificity. Violin representation of median and interquartile range. **(B)** Correlation matrix between glycan expression by tumor cells (assessed by lectin fixation) and CLR expression on tumor-infiltrating DCs from the corresponding patients (n=4-5 matched samples). Only CLRs found to be modulated in tumor infiltrates compared to control tissue are analyzed. Spearman correlation. * p<0.05.

To further assess the underlying mechanism behind the altered CLR expression profiles, we analyzed glycan motifs on tumor cells by performing lectin arrays of primary melanoma tumor cells derived from patients using GLYcoPROFILE™ technology, and executed correlation between specific glycan motifs and expression of the CLR that we found modulated on tumor-infiltrating DC subsets within corresponding samples. Melanoma tumor glycocode was assessed by investigating lectins fixation, indicators of the expression levels of the corresponding glycans (**Supplementary Table 3**). The glycoprofile of primary melanoma tumor cell lines revealed high level of Man, Fuc and GlcNAc motifs (as shown by high fixation of the lectins ConA, PSA, BC2LA, RPL-αMan and WGA) (**Figure 5A**). These motifs are recognized by Dectin-1 (specific for β-glucans), DCIR, DC-SIGN and CD206 (specific for Man, Fuc and GlcNAc motifs). Strikingly, these CLRs (Dectin 1, DCIR, DC-SIGN and CD206) are the one that were the most modulated on tumor-infiltrating DC subsets from melanoma patients (**Figures 1**–**3**). Notably, correlation matrix between glycan expression by tumor cells (assessed by lectin fixation) and CLR expression on tumor-infiltrating DCs from corresponding patients (**Figure 5B**; **Supplementary Table 4**) highlighted strong link between specific glycans and corresponding CLR on DCs within the tumor of melanoma patients. Indeed, frequency of tumor-infiltrating DCIR cDC2s positively correlated with level of WGA fixation by tumor cells, therefore levels of GlcNAc motifs. Tumor-infiltrating Dectin1+ cDC1s were linked with PSA and WGA fixation, thus with Glc motifs on tumor cells. Frequency of tumor-infiltrating DC-SIGN+ cDC2s was negatively linked with lectin recognizing Man motifs (GNA, RPL-αMan). These data strongly support that altered CLR expression profiles on DCs’ subsets from melanoma patients are linked with specific glycan patterns on melanoma tumor cells, but could also be related to the presence of necrotic cells or other released factors such as DAMPs.

Altogether, these results demonstrated that melanoma tumor cells specifically alter CLR expression profiles of healthy DC subsets, both directly and indirectly through soluble factors depending on the DC subset, suggesting that melanoma tumor cells displayed and released glycans or DAMPs that may interact with DCs through CLR molecules.

### Modulation of CLR profiles on DC subsets correlates with disease progression and clinical outcome of melanoma patients

**Figure 6:**
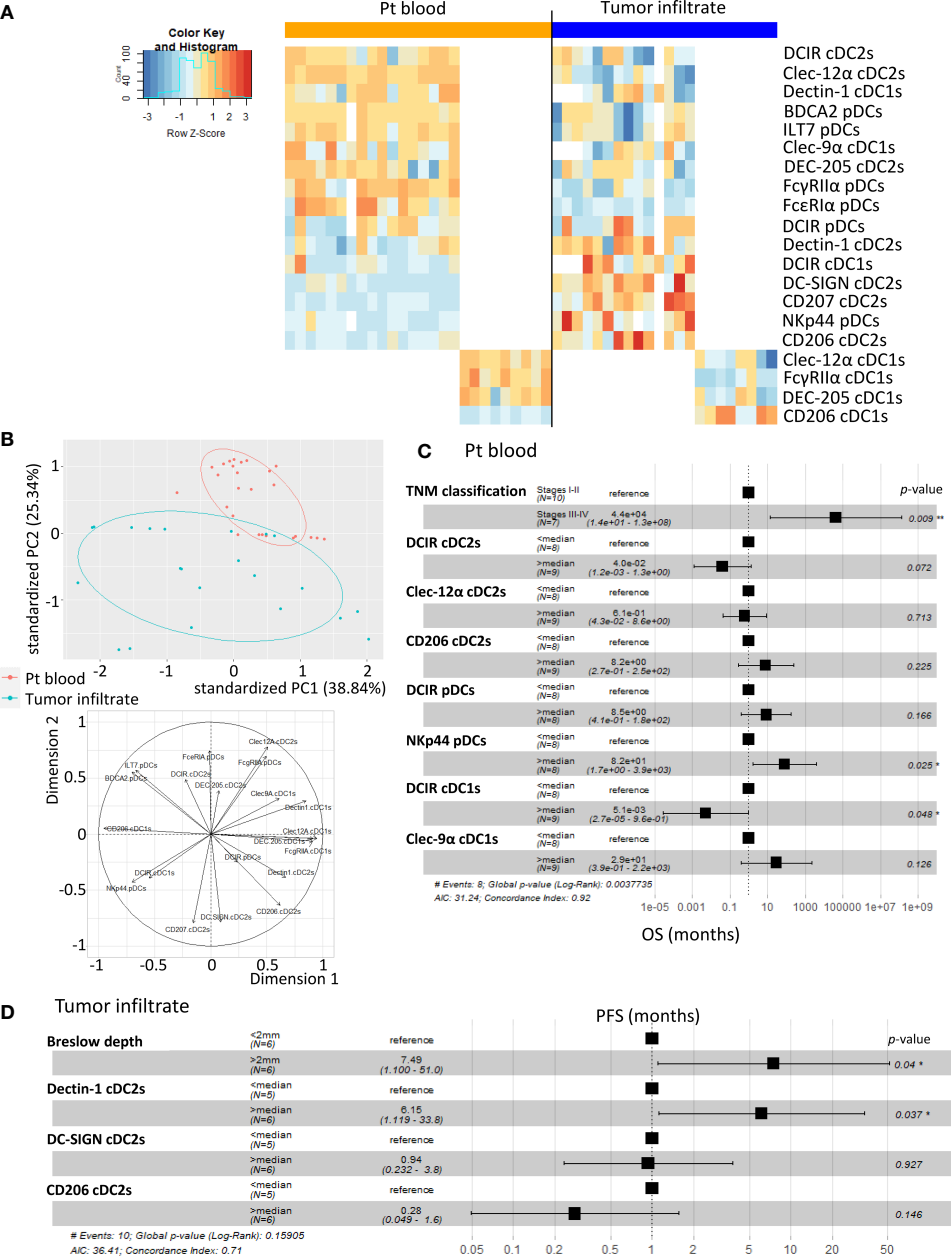
A specific CLR profile on circulating and tumor-infiltrating DC subsets is associated with melanoma progression. Multiple regressions were performed to study the potential link between CLR expression by DCs subsets (derived from PBMC and tumor-infiltrating cells from melanoma patients) and tumor progression. **(A)** Heat map based on the percentage of expression of the whole panel of CLRs studied on each DC subset from patients’ blood (n= 26) and tumors (n= 21) (DCIR, Dectin-1, Clec-9α, DEC-205, Clec-12α, FcγRIIα and CD206 on cDC1s; DCIR, Dectin-1, Clec-9α, DEC-205, Clec-12α, FcγRIIα and CD206 on cDC1s; and DCIR, NKp44, ILT7, FcγRIIα, FcϵRIα and BDCA2 on pDCs). **(B)** PCA based on CLR expression by DC subsets from patient’s blood and tumors (including graph of variables). **(C)** Hazard ratios from comparative overall survival (OS) (from sampling time) of TNM classification and expression of specific CLRs (previously seen modulated in patients’ blood when compared to control) by circulating DC subsets of melanoma patients (n= 17). For CLR expression on DC subsets, groups were separated using the median percentage of CLR expressing DC subsets from patients’ blood. **(D)** Hazard ratios from comparative progression-free survival (PFS) (from diagnostic time) of Breslow depth and expression of specific CLRs (Dectin-1, DC-SIGN or CD206) (previously seen modulated in tumors when compared to control) by tumor-infiltrating cDC2s (n= 11 to 12). For CLR expression on DC subsets, groups were separated using the median percentage of CLR expressing cDC2s from tumors. * P-value < 0.05.

**Figure 7:**
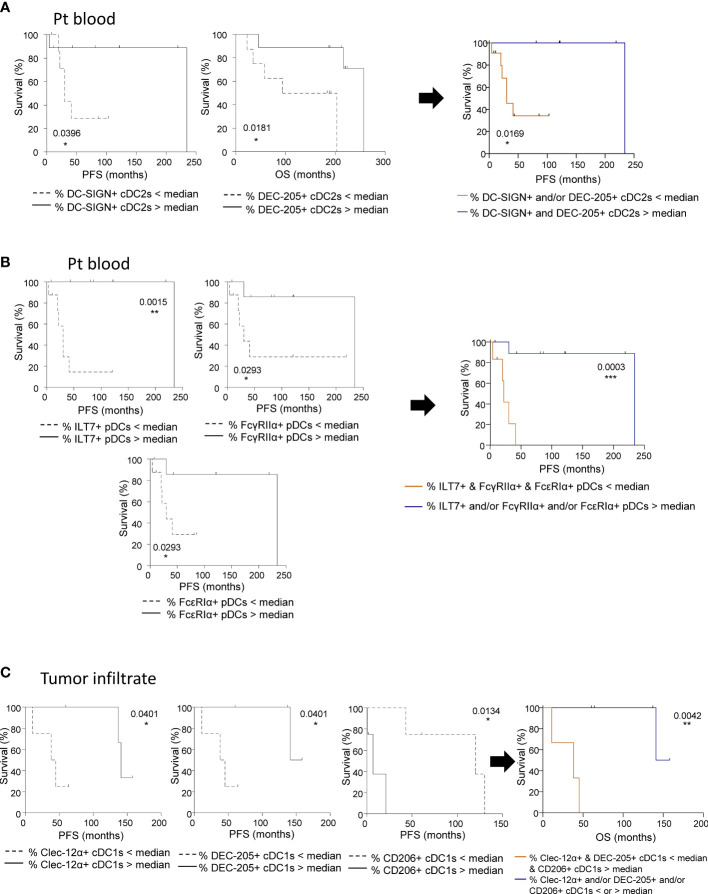
CLR expression by circulating and tumor-infiltrating DC subsets from melanoma patients dictates clinical outcomes. Survival analyses were performed to study the potential correlation of CLR expression by DCs subsets (derived from PBMC and tumor-infiltrating cells from melanoma patients) and clinical outcome of the patients. **(A)** Comparative PFS and OS (from diagnostic time) of patients with low or high levels of circulating DC-SIGN^+^ cDC2s (left panel), DEC-205^+^ cDC2s (middle panel) and DC-SIGN^+^ and/or DEC-205^+^ cDC2s (right panel). Groups were separated using the median percentages of circulating DC-SIGN^+^ cDC2s (2.84%) and/or circulating DEC-205^+^ cDC2s (79.85%) (n= 6 to 11 patients/group). **(B)** Comparative PFS and OS (from diagnostic time) of patients with low or high levels of ILT7^+^, FcγRIIα^+^, or FcϵRIα^+^ pDCs (left panels), and ILT7^+^ and/or FcγRIIα^+^ and/or FcϵRIα^+^ pDCs (right panel). Groups were separated using the median percentages of circulating ILT7^+^ (92.33%), FcγRIIα^+^ (81.60%), and/or FcϵRIα^+^ (57.70%) pDCs (n= 6 to 8 patients/group). **(C)** Comparative PFS and OS (from diagnostic time) of patients with low or high levels of tumor-infiltrating Clec-12α^+^ (left panel) or DEC-205^+^ (middle left panel) or CD206^+^ (middle right panel) cDC1s, and Clec-12α^+^ and/or DEC-205^+^ and/or CD206^+^ cDC1s (right panel). Groups were separated using the median percentages of tumor-infiltrating Clec-12α^+^ (93.83%), DEC-205^+^ (38.15%) and/or CD206^+^ (20.64%) cDC1s (n= 3 to 5 patients/group). **(A-C)** Comparisons using Log-rank test. * P-value < 0.05, ** P-value < 0.001, *** P-value < 0.0001.

To evaluate the clinical relevance of perturbed CLR expression profiles on circulating and tumor-infiltrating DC subsets of melanoma patients, we studied their association with the clinical outcome of patients by performing multiple regressions (Cox regression; Kaplan-Meier; **Supplementary Tables 5** and **6** respectively). The global view of patient’s samples, through a heat map illustration, highlighted distinct patterns of CLR expression on tumor-infiltrating DCs when compared to circulating DCs (**Figure 6A**), strongly suggesting that the tumor microenvironment triggers potent perturbations of CLR profiles on DCs. Furthermore, DCs from patient’s blood and tumors could be differentiated by their CLR expression profile since both groups were located in distinct areas of the PCA analysis (based on PC1 and PC2) (**Figure 6B**). Yet, no differences of CLR expression profiles on circulating DCs were found depending on the disease stage (determined by TNM classification; **Supplementary Figure 8A**). To assess the prognostic impact of CLR expression profiles on DCs, we further examined the hazard ratios of modulated CLRs on DCs combined with some major prognosticators of melanoma progression (such as TNM classification and Breslow depth). As expected, patients with advanced stages or with initial high Breslow depth were more likely to undergo shorter survival or early relapse in our cohort (**Figures 6C, D**; **Supplementary Figure 8B**). Notably, patients with higher levels of CD206 MFI on cDC2s or higher frequencies of NKp44^+^ pDCs in their blood were more likely to have worse clinical outcome, as indicated by their shorter overall survival (OS), than patients with lower levels of CD206 on cDC2s (HR = 41.34 and *P*-value = 0.011; **Supplementary Figure 8B**) or fewer NKp44^+^ pDCs (HR = 8.2 and *P*-value = 0.025; **Figure 6C**). Conversely, patients with high frequencies of circulating DCIR^+^ cDC1s were more likely to experience better clinical outcome, as indicated by their longer OS, than patients with low frequencies (HR = 0.0051 and *P*-value = 0.048; **Figure 6C**). Furthermore, patients with higher frequencies of tumor-infiltrating Dectin-1^+^ cDC2s were 6 times more likely to undergo worse outcome, as indicated by shorter progression-free survival (PFS), than patients with low frequencies (HR = 6.15 and *P*-value = 0.037; **Figure 6D**). Such observations revealed that frequencies of CLR-expressing DCs in blood and tumor could be independent prognosticators of the patients’ clinical outcome. We further analyzed the link between CLR expressions on DC subsets and patients’ clinical outcomes by performing Kaplan-Meier survival analyses based on CLRs whose expression was either perturbed compared to controls or displayed heterogeneity within patients. Indeed, higher frequencies of circulating DC-SIGN^+^ or DEC-205^+^ cDC2s were linked with better PFS or OS respectively (**Figure 7A**; left and middle panels), and high frequencies of circulating cDC2s expressing simultaneously both CLRs strongly correlated with better PFS (**Figure 7A**; right panel). Additionally, high levels of DCIR expression by circulating DCIR^+^ cDC2s predicted better clinical outcome, whereas high levels of CD206 expression by circulating CD206^+^ cDC2s were associated with worse OS in melanoma patients (**Supplementary Figure 9A**). Strikingly, we also found that higher proportions of circulating ILT7^+^, FcγRIIα^+^ and/or FcϵRIα^+^ pDCs were linked with a longer PFS (**Figure 7B** left), and patients whose pDCs expressed simultaneously high levels of these three CLRs strongly underwent better PFS (**Figure 7B**; right panel). Moreover, lower MFI for Dectin-1 by circulating Dectin-1^+^ cDC1s was linked with a worse clinical outcome (**Supplementary Figure 9B**), especially in the concomitant presence of circulating DCIR^+^ cDC2s with low MFI for DCIR (**Supplementary Figure 9C**). Interestingly, simultaneously higher frequencies of DC-SIGN^+^ cDC2s and ILT7^+^ or FcγRIIα^+^ pDCs were associated with better outcome (**Supplementary Figure 9C**). Yet, lower frequencies of simultaneously DEC-205^+^ cDC2s and ILT7^+^ pDCs were linked with worse OS in patients (**Supplementary Figure 9C**). Strikingly, higher frequencies of tumor-infiltrating Clec-12α^+^ or DEC-205^+^ cDC1s were associated with a better clinical outcome as indicated by longer PFS in patients, whereas higher proportions of tumor-infiltrating CD206^+^ cDC1s were linked with a poor survival even worse when associated with low frequencies of tumor-infiltrating Clec-12α^+^ and DEC-205^+^ cDC1s (**Figure 7C**). Additionally, higher MFI for CD206 or FcγRIIα by tumor-infiltrating CD206^+^ or FcγRIIα^+^ cDC1s respectively were linked with a poor clinical outcome (**Supplementary Figure 9D**). Thus, even if based on rather small sample sizes, specific CLR expression profiles or combinations of CLR patterns on DC subsets in melanoma patients were associated with disease progression and clinical outcome.

### CLR expression on DC subsets is linked with DCs’ activation status and functionality in melanoma patients

**Figure 8:**
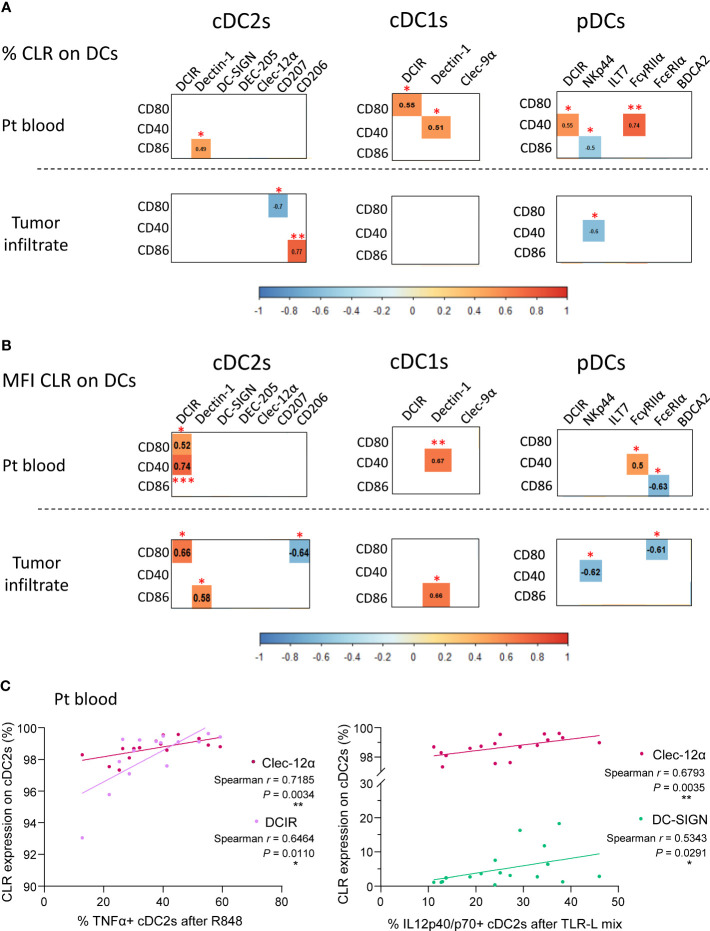
CLR expression by circulating and tumor-infiltrating DC subsets correlates with DCs’ activation status and functionality in melanoma patients. Spearman correlation were performed to assess the link between CLR expression by DC subsets and DCs’ basal activation status or cytokine production after TLR triggering. **(A)** Correlation matrix between frequencies of CLR-expressing DC subsets (cDC2s, cDC1s and pDCs) and their basal activation status (frequencies of DC subsets expressing CD80, CD40 and CD86) in patient blood (n= 16 to 17; upper panels) and tumor infiltrate (n=11 to 13; bottom panels). **(B)** Correlation matrix between comparative expression levels (MFI) of CLR-expressing DC subsets and their basal activation status (frequencies of DC subsets expressing CD80, CD40 and CD86) in patient blood (n=16 to 17; upper panels) and tumor infiltrate (n= 11 to 13; bottom panels). **(A, B)** Only spearman correlations with significant *P*-values (<0.05) were showed. *P-value ≤ 0.05, **P-value ≤ 0.01, ***P-value ≤ 0.001. **(C)** Spearman’s correlation of frequencies of TNFα^+^ (left panel) or IL-12p40/p70^+^ (right panel) cDC2s upon TLR triggering (R848 or TLR-L mix) and basal CLR expression (Clec-12α, DCIR or DC-SIGN) by circulating cDC2s in melanoma patients (n=15 to 17).

As we previously highlighted major alterations of circulating and tumor-infiltrating DCs’ features in melanoma patients, we further explored whether CLR expression profiles on DC subsets correlated with DCs’ activation status and functionality in melanoma patients. We first ensure that DCs’ functionality was not disturbed between fresh and frozen samples, allowing combining frozen samples from all groups at the same time (**Supplementary Figure 2B**). We performed Spearman correlations between CLR expression and basal activation status or cytokine production by DC subsets in matched samples (**Supplementary Tables 7** and **8** respectively). Data regarding DCs’ activation status and functionality in melanoma patients derived from the same samples as the current study, and were previously published (7). Regarding DC basal activation status (studied by CD80, CD40 and CD86 expression), we found positive correlations between proportions and/or MFI of circulating DCIR^+^ or Dectin-1^+^ cDC2s or cDC1s and proportions of CD80-, CD40- or CD86-expressing cDCs (**Figures 8A, B**). Expression of co-stimulatory molecules by pDCs positively correlated with circulating DCIR^+^ or FcγRIIα^+^ pDCs, but negatively correlated with circulating or tumor-infiltrating NKp44^+^ or FcϵRIα^+^ pDCs (**Figures 8A** and 8B). Furthermore, an additional negative correlation was found in tumors between CD80^+^ and CD207^+^ cDC2s (**Figure 8A**). Strikingly, when investigating correlations between CLR expression and cytokine production by circulating DCs, proportions of Clec-12α^+^, DCIR^+^ or DC-SIGN^+^ cDC2s positively correlated with proportions of TNFα^+^ and/or IL12p40/p70^+^ cDC2s upon TLR stimulation (**Figure 8C**). Thus, specific CLR expression profiles on DC subsets correlated with unique DCs’ features in melanoma patients, suggesting that melanoma tumor may shape DCs’ features by exploiting the plasticity of the CLR machinery.

## Discussion

**Figure 9:**
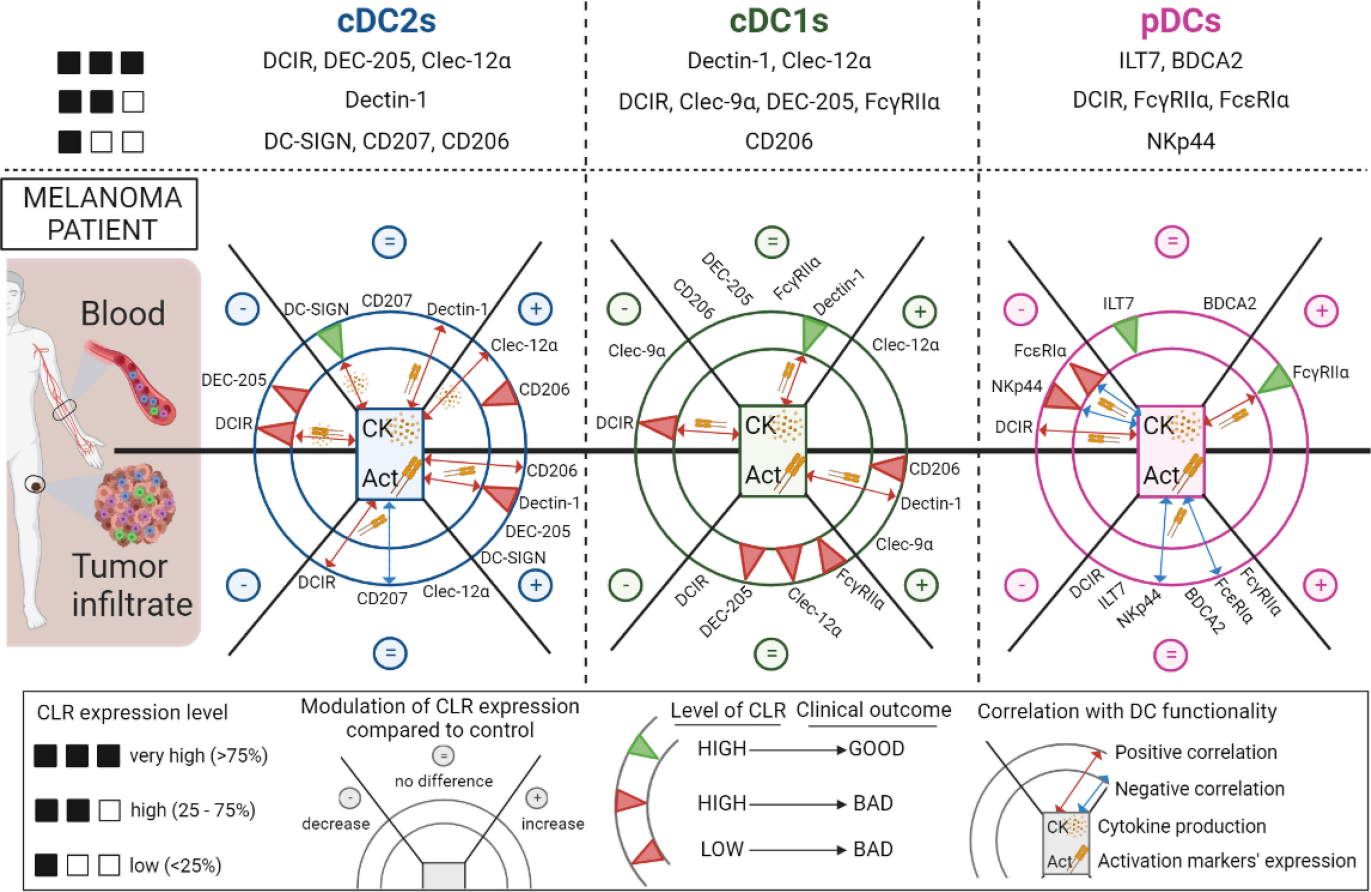
Overview of the modulations of CLR expression profiles on circulating and tumor-infiltrating DC subsets in melanoma patients, their correlation with DCs’ features and their clinical relevance. Overview of the CLR expression pattern on cDC2s, cDC1s and pDCs (upper panels). Modulations of CLR expression (decrease (–), no difference (=) and increase (+)) on cDC2s (left panel), cDC1s (middle panel) and pDCs (right panel) in patients’ blood (upper half-circles) and tumor infiltrate (bottom half-circles) when compared to controls are represented in the external portions of the circles representing each DC. The impact of CLR expression levels by DCs on patients’ clinical outcome are symbolized as green or red triangles (good or bad outcome respectively) in the internal concentric circles of each DC subset. Red or blue double arrows (positive or negative correlations respectively), located in the inner part of each DC subset, symbolized correlations between CLR expression and DC functionality (basal activation status or cytokine production upon TLR stimulation). This figure has been created using BioRender science illustration tool.

Recognition of tumor glycans by CLRs expressed by DCs together with subsequent signaling cascades are crucial to shape antitumor immunity, and decisive in the orientation of the response. Yet the status of the CLR machinery on DCs in melanoma remained largely unknown. For the first time, we depict CLR expression patterns on circulating and tumor-infiltrating cDC1s, cDC2s and pDCs, and unravel that melanoma tumor cells may exploit CLR pathways to hijack DC subsets and escape from immune control (**Figure 9**, Graphical summary). Our study opens the way for innovative strategies exploiting glycan-CLR interactions to restore efficient antitumor immunity.

We highlighted for the first time that the CLR repertoire of circulating and tumor-infiltrating cDC2s, cDC1s, and pDCs is strongly perturbed in melanoma patients. The choice of studied CLRs was based on the expression of the corresponding glycans on melanoma tumor cells, therefore orientating our analysis on receptors for Man, Fuc and GlcNAc motifs. Major CLR modulations could be observed for ITAM-coupled CLRs such as Dectin-1 and Clec-9α, ITIM-coupled CLRs in particular DCIR and Clec-12α, endocytic CLRs especially CD206, DEC-205 and DC-SIGN, and adaptors as FcγRIIα and FcεRIα. Notably, the expression of some CLRs was linked with DCs’ activation status and functionality; either positively for DCIR, Dectin-1, Clec12α, DC-SIGN and CD206 on cDCs or negatively for NKp44 and FcεRIα on pDCs. This suggested that interactions between tumor glycans and CLRs may influence DCs’ response and dictate DCs’ features both in blood and within tumor. Moreover, the specific pattern of CLR expression by tumor-infiltrating DCs compared to circulating DCs strongly suggest that melanoma harbored glycan motifs able to interact with DCs through CLRs, especially mannose and fucose structures (61), as highlighted by the melanoma glycocode. Such differential CLR profiles could also be linked with the differential activation status of DC subsets. We previously highlighted that cDC2s, cDC1s and pDCs from the tumor microenvironment displayed higher levels of co-stimulatory molecules compared to the circulating DC subsets (7), potentially explaining distinct CLR expression patterns observed in blood and tumor of melanoma patients. Altogether, such observations indicated that tumor-derived carbohydrates may interact with the CLR machinery and modulate DCs’ function, hence modifying the outcome of the response by exploiting glycan-CLR interactions.

Among changes, DCIR was downregulated on all circulating DC subsets. DCIR recognizes mannose/fucose-based glycans and signals through ITIM motifs. It has been shown that cross-linking DCIR with agonists antibodies inhibited TLR8-driven production of IL-12 by cDCs and TLR9-induced IFNα by pDCs (45, 62). Such modulation may reflect engagement of DCIR by melanoma-derived carbohydrates resulting in further functional inhibition of DCs. Targeting DCIR can however lead to antigen processing and presentation for efficient T-cell priming (63), which offers the opportunity to simultaneously target all three DC subsets to restore antitumor response.

Interestingly, Dectin-1 expression increased on tumor-infiltrating cDC2s and cDC1s in melanoma patients. Dectin-1 recognizes β-1,3-glucans expressed by a broad range of fungal pathogens and bacteria, and also endogenous factors such as galectin-9 or N-glycans on tumor cells. A high signaling flexibility has been observed for Dectin-1 in response to the same ligand, potentially depending on the amino acid sequence of the CLR responsible for ligand valency (64). Upon engagement, Dectin-1 uses the Syk/CARD9 pathway to mediate NF-kB activation, promote cytokine production, and drive the development of Th1 and cytotoxic CD8^+^ T cells, hence orchestrating antitumor immunity (48, 65). Besides, targeting Dectin-1 with β-glucan triggered increased T-cell infiltration within tumor together with reduced Treg and MDSCs (66), sustaining advantages of targeting Dectin-1 in melanoma.

Besides, many endocytic CLRs were found to be upregulated in tumor infiltrating cDC2s and cDC1s in melanoma patients. CD206, DEC-205 and DC-SIGN bind to mannosylated and fucosylated residues, and led to the internalization of bound ligands and presentation of antigens. Targeted delivery of tumor antigens to DEC-205 and DC-SIGN with appropriate adjuvants prevented tumor development in mouse models, especially for melanoma (67). DC-SIGN engagement by sialyl-Lewis motifs activates and enhances the maturation of DCs leading to the enrichment of antigen-specific IFNγ production and CTL response, and the suppression of tumor growth in mice (68). Glycan-modified liposomes targeting DC-SIGN improved antigen-presentation by DCs and boosted CD4 and CD8 T cell responses (69). However, CD206 can also mediate the immuno-suppressive function of immune cells. Indeed, in ovarian carcinoma, engagement of CD206 on tumor-associated macrophages by tumor-derived mucins promoted cytokine production toward an immune suppressive profile (70). In addition, Clec-9α displayed major perturbation on circulating and tumor infiltrating cDC1s of melanoma patients. It recognizes necrotized cells *via* exposed actin filaments (40) and mediate antigen cross-presentation to CD8 T cells (17). Modulation of these endocytic CLRs may reflect an increased activity of phagocytosis and cross-presentation by tumor-infiltrating cDCs in melanoma.

Modulation of adaptors FcγRIIα and FcεRIα on circulating pDCs may reflect the strong mobilization of CLRs that recruit these adaptors upon their engagement, especially ILT7 and BDCA2. These two CLRs are known to strongly inhibit the production of type I IFN and pro-inflammatory cytokines by pDCs upon respectively ligation of BST2 (71) or antibody triggering (49). Interestingly, BDCA2 also binds to galactose-terminated glycans (epitope Galβ1–3/4GlcNAcβ1–2Man through its extracellular CRD domain) (72). NKp44 was also perturbed on pDCs. It has been identify that NKp44 cross-linked Proliferating Cell Nuclear Antigen (PCNA) on tumor cells, and its triggering by agonists led to inhibition of IFNα production in response to CpG (52). Very interestingly, blocking the NKp44-PCNA interaction resulted in inhibition of tumor growth including melanoma in mouse models (73). Thus interaction between NKp44 on pDCs and PCNA on tumor cells may explain pDC dysfunction in melanoma (5, 7).

We demonstrated that perturbations of CLR expression profiles could be triggered directly by melanoma cells but also indirectly through melanoma-derived supernatants, suggesting that melanoma tumor cells displayed and released glycans that may interact with DCs through CLR molecules. Indeed, it has been shown that melanoma tumor cells exhibited a high ganglioside diversity (74), displayed alterations in the glycosylation pattern of glycoproteins and glycolipids (57, 58), and perturbations of enzymes involved in glycosylation/deglycosylation processes (56). The impact of tumor-associated glycans on DCs in the context of melanoma remained unexplored. Yet, interactions between tumor carbohydrates and CLRs on myeloid cells including DCs have been highlighted in other tumor models. In colorectal cancer Lewis glycans on CEA drove impairment of monocyte-derived (mo) DCs’ function upon binding of DC-SIGN and subsequent Th2/Treg responses (54), whereas in ovarian adenocarcinoma sialic acids on MUC1 triggered antitumor activities of macrophages through Dectin-1 and CD206 (75). Altogether, these studies suggest that interactions between tumor-associated carbohydrates antigens (TACAs) and CLRs on DCs are crucial to shape subsequent immune responses, and justify further investigations in melanoma to better understand and exploit the glycan/CLR axis.

We examined the CLR profile of cDC2s, cDC1s and pDCs as we previously demonstrated that these subsets exhibited dysfunctional features in melanoma patients (7, 8). We observed that all three subsets displayed a perturbation of their CLR expression profile, but still highlighted differences between DCs. Based on analyses of both circulating and tumor-infiltrating DC subsets in patients and healthy DCs, it turns out that cDC2s were more affected within tumor microenvironment despite small perturbations in blood, pDCs were modulated mostly in blood circulation, and cDC1s perturbed in both blood and tumor. These observations are in accordance with the fact that the CLR profile on healthy DCs was perturbed mostly by tumor cells for cDC2s, essentially by tumor-derived supernatants for pDCs, and by both for cDC1s. This also highlighted the additional level of tremendous complementarity between DC subsets according to their CLR equipment. DCs can thus recognize a wide diversity of tumor carbohydrates, which combine to the downstream diversity of signaling pathways, offers huge opportunities to fine-tune immune responses, and this complexity may allow multiple ways for DCs to prevent their subversion by tumor cells. Further understanding of CLR signaling pathways and immune outcomes in each DC subset together with the tumor glyco-code is crucial to deeply decipher the complexity of interactions between cancer cells and immune cells within the tumor microenvironment, and exploit the glycan/CLR axis in immunotherapy.

CLR profiling revealed some discrepancies and opposite regulation for a specific CLR between blood and tumor (Clec-9α on cDC1s) or between DC subsets (Clec-12α and CD206 on circulating cDC2s and cDC1s). As CLR expression pattern is a dynamic process and depends on the presence of ligands, a possible competition may exist between DC subsets, the capture of patterns at a specific time may be difficult to interpret. Moreover, many CLRs are major players for both antitumor immunity and immune evasion. In addition, single nucleotide polymorphism (SNPs) have been outlined in genes coding CLRs and could constitute a risk factor for cancer development, as demonstrated for DC-SIGN for which SNPs in the promoter modulate the susceptibility of developing colorectal cancer (CRC) (76). Further exploration on the influence of the cell type, CLR expression levels, signaling pathways induced, the nature of the ligand and the role of the microenvironment on CLR signaling are needed to have a better understanding of how CLRs affect immunological outcomes.

Based on their pivotal role in shaping and regulating immune responses, CLRs harbor promising potential for therapeutic developments. Manipulating the CLR machinery holds considerable promises for cancer therapy. The modulation and/or targeting of DCs through CLR emerged as an interesting approach to induce antitumor immunity, using antigen-coupled anti-CLR antibodies, CLR ligands or carbohydrate-coated antigen-containing nanoparticles (38, 77–79). Indeed, glycans coupled to antigens can efficiently target DCs through DC-SIGN, leading to antigen internalization, cross-presentation and subsequent elicitation of tumor-specific CD4^+^ and CD8^+^ T-cell responses (80). Modification of gp100/MART1 with mannose or Lewis structures enhanced targeting to moDCs and antigen presentation to specific T cells through DC-SIGN (81, 82). Tumor antigen coupled to anti-DEC-205 or anti-DC-SIGN antibodies combined with a strong DC activator (TLR-L) triggered a potent antigen-specific immune activation resulting in clearance of the tumor (83). Targeting antigen to cDC1s through anti-Clec-9α antibody elicited specific CD4^+^ and CD8^+^ T-cell responses and promoted antitumor responses (84, 85). Besides, agonists or antagonists of CLRs are also promising therapeutic reagents for cancer immunotherapy, as mobilization of CLRs upon ligand recognition initiate signaling cascades that positively or negatively regulate immune responses. Activation of ITAM-based CLRs should support the development of protective immunity. Conversely, anti-inflammatory pathways driven by ITIM-based CLRs could help prevent inflammation-induced cancers. β-glucans (Dectin-1 agonist) exhibited antitumor activities in several mouse tumor models (86) by reprograming tumor-infiltrating DCs to secrete IL-12p70 favoring Th1 response (87). In human, β-glucans are currently being tested in many clinical trials, especially in combination with conventional chemotherapy with encouraging outcomes. CLR ligands can therefore be used as adjuvants to redirect immune responses. Thus, CLRs can mediate both antigen delivery and immune orientation. All these therapeutic exploitations required to first depict the CLR expression pattern of DCs in patients and to master the subsequent functional orientation of DCs. For example, the poor expression of DC-SIGN and CD206 on circulating cDC2s in melanoma patients questions the use of these CLRs for therapeutic exploitation, and emphasizes the need to perform pre-clinical studies on *in situ* DCs and not *in vitro* made DCs, which display a different CLR expression pattern. The exciting potential of targeting CLRs to restore efficient antitumor responses supports further investigations in the field.

The present work sheds light on a new pathway used by melanoma to hijack DC subsets and escape immunity. We unraveled that cancer cells may exploit the CLR machinery of DCs to induce immunosuppressive signaling and evade from immunity. CLRs are key immune checkpoints to shape and control immunity upon sensing tumor carbohydrates. Further exploration of the glycan/lectins circuits is crucial to understand tumor-induced immune evasion and design future innovations to properly reshape antitumor immunity by exploiting the CLR machinery.

## Data availability statement

The original contributions presented in the study are included in the article/**Supplementary Material**. Further inquiries can be directed to the corresponding author.

## Ethics statement

The studies involving human participants were reviewed and approved by the French Blood Service’s (EFS-AuRA) Institutional Review Board and the ethics committee of Grenoble University Hospital (CHU-Grenoble) and declared under the reference #DC-2008-787. Written informed consent was acquired from all participants prior to their participation in this study.

## Author contributions

CA conceived the project and supervised the study; CA, ES designed the experiments and wrote the manuscript; ES, CA, BR, LL performed the experiments and analyzed the data. SM, JC, FdF provided patient material and clinical data, and expertise with clinical interpretation of the data; JVG, NBV, LC, BR, LL provided research input and contributed to data interpretation; LC, SM, NBV contributed to manuscript editing. All authors contributed to the article and approved the submitted version.

## Funding

This work was supported by the Etablissement Français du Sang (EFS) AuRA, Ligue contre le Cancer, the Société Française de Dermatologie (SFD), GEFLUC, Fondation BMS, and Université Grenoble Alpes (UGA).

## Acknowledgments

We thank Dr D. Legrand and her staff at EFS Auvergne Rhone-Alpes for providing healthy volunteers’ blood samples. We acknowledge the surgeons and anatomo-pathology Department from CHU Grenoble Alpes for providing tumor samples especially Pr Nicole Pinel and Pr Nathalie Sturm. We thank Pr Riggini and his team from CHU Grenoble Alpes for providing tonsils. We thank Cyrielle Chauviere and Leana Corneloup for their help with some experiments. We are grateful to M. Pezet, A. Grichine, and P. Marche for access to the cytometry platform; and all the volunteers and patients who agreed to participate in this study. We thank Pr Franck Fieschi for stimulating discussions and precious advices.

## Conflict of interest

Authors BR and LL were employed by company GLYcoDiag.

The remaining authors declare that the research was conducted in the absence of any commercial or financial relationships that could be construed as a potential conflict of interest.

## Publisher’s note

All claims expressed in this article are solely those of the authors and do not necessarily represent those of their affiliated organizations, or those of the publisher, the editors and the reviewers. Any product that may be evaluated in this article, or claim that may be made by its manufacturer, is not guaranteed or endorsed by the publisher.
