# Supplementary material for: Unique CLR expression patterns on circulating and tumor-infiltrating DC subsets correlated with clinical outcome in melanoma patients
Source: Front Immunol. 2022 Oct 24;13:1040600. doi: 10.3389/fimmu.2022.1040600 (PMC9638162; doi:10.3389/fimmu.2022.1040600)
Supplement: Supplementary file 1 [file DataSheet_1.pdf]

Supplementary Material

1 Supplementary Figure legends

<u>Supplementary figure 1:</u> **Multi-parametric flow cytometry approach to depict CLR expression on the three major DC subsets**

Representative flow cytometry plots from HD blood. (A) DC subsets' gating strategy. By using antibodies targeting specific surface markers, we depicted the three DC subsets from the same sample and studied their respective CLR expression. FSC-A and SSC-A parameters allowed to exclude cell debris, and then single cells were gated with FSC-A and FSC-H parameters. The exclusion of dead cells was done using a Live and Dead cell staining. Within $CD45^+$ cells, pDCs were depicted has HLA-DR^+BDCA4^+ cells. Furthermore, amongst Lin^- HLA-DR^+ cells, CD11c and BDCA1 or BDCA3 markers allowed to define cDC2s and cDC1s respectively. Representative flow cytometry plots from HD blood. (B) Representative dot plots depicting CLR expression by the three DC subsets in blood (HD, Pt) and tissues (non-tumor and metastatic melanoma). DC-SIGN, Clec9α and BDCA2 are shown as examples.

<u>Supplementary figure 2</u>: **CLR expression profiles and DCs' functionality are similar in fresh and frozen samples**

CLR expression (A) and DCs' functionality (B) were assessed on circulating DCs' subsets on matched fresh and frozen PBMC. Fresh and frozen PBMC from healthy donors were labelled with specific antibodies to depict CD11c+BDCA1+ cDC2s, $CD11c^+BDCA3^+$ cDC1s and $CD11c^-BDCA4^+$ pDCs amongst alive CD45+Lin-HLA-DR+ cells and their CLR expression was assessed by flow cytometry. To evaluate pDCs' functionality, fresh and frozen PBMC from melanoma patients were stimulated for 5h with CpG_A, and IFNα and IP10 secretion was

assessed by intracellular cytokine staining within pDCs. A/ Comparative CLR expression on circulating cDC2s, cDC1s and pDCs from fresh and frozen samples (n=6 matched samples). B/ Comparative viability, frequency of pDCs as well as IFNα- and IP10-secreting pDCs from fresh and frozen samples (n=6 matched samples).

Supplementary figure 3: cDC2s from the blood and tumor of melanoma patients harbor specific CLR profile

Circulating and tumor-infiltrating immune cells from melanoma patients and controls were labelled with specific antibodies to depict $CD11c^{+}BDCA1^{+}$ cDC2s amongst alive $CD45^{+}Lin^{-}HLA\text{-}DR^{+}$ cells and their CLR expression was assessed using flow cytometry. (A) Heat map based on the comparative expression levels (MFI) of DCIR, Dectin-1, DC-SIGN, DEC-205, Clec-12α, CD207 and CD206 on cDC2s derived from patients' blood (n=17) and tumors (n=13) and controls' blood (n=26) and tissue (n=9). (B) Principal component analysis (PCA) based on MFI of CLR expression on cDC2s of the four groups studied (including graph of variables). (C) MFI of DCIR, Dectin-1, DC-SIGN, DEC-205, Clec-12α, CD207 and CD206 on cDC2s from the blood of healthy donors (HD, open circles, n=26) and melanoma patients (Pt, filled circles, n=17), and tissue control (open triangles, n=9) and tumor infiltrates of melanoma patients (filled triangles, n=13). Results are expressed as MFI of the CLR amongst CLR-positive cDC2s. Only significant statistics are shown on the graphs. Bars indicate median. *P*-values were calculated using Mann-Withney (dashed lines) and Kruskal-Wallis (full lines) non parametric tests. **P-value ≤ 0.01, ***P-value ≤ 0.001, ****P-value ≤ 0.0001.

Supplementary figure 4: The age do not affect CLR expression profiles by DCs' subsets of melanoma patients

Circulating (A) and tumor-infiltrating (B) immune cells from melanoma patients were labelled with specific antibodies to depict CD11c+BDCA1+ cDC2s, $CD11c^{+}BDCA3^{+}$ cDC1s and $CD11c^{-}BDCA4^{+}$ pDCs amongst alive CD45+Lin-HLA-DR+ cells and their CLR expression was assessed by flow cytometry. Groups were separated by the median age of the cohort (white bars: age<median; grey bars: age>median). A/ Comparative CLR expression profiles by circulating cDC2s, cDC1s and pDCs between "young" (n=8) and "older" (n=9)

patients. Median age = 49 years. B/ Comparative CLR expression profiles by tumor-infiltrating cDC2s, cDC1s and pDCs between "young" (n=7) and "older" (n=7) patients. Median age = 59 years. Bars indicate mean+/-SEM. *P*-values were calculated using Mann-Withney non parametric tests.

<u>Supplementary figure 5:</u> Circulating and tumor-infiltrating cDC1s display specific CLR profile in melanoma patients

Circulating and tumor-infiltrating immune cells from melanoma patients and controls were labelled with specific antibodies to depict $CD11c^{+}BDCA3^{+}$ cDC1s amongst alive $CD45^{+}Lin^{-}HLA\text{-}DR^{+}$ cells and their CLR expression was assessed using flow cytometry. (A) Heat map based on the MFI of DCIR, Dectin-1, Clec-9α, DEC-205, Clec-12α, FcγRIIα and CD206 on cDC1s derived from patients' blood (n=26) and tumors (n=18), and controls' blood (n=31) and tissue (n=9) (juxtaposition of two non-supervised clustering performed independently on the two sub-cohorts). (B) Graph of variables for the PCA based on CLR expression on cDC1s of the four groups studied for the two distinct data sets (DCIR, Dectin-1, Clec-9α in the left panel; and DEC-205, Clec-12α, FcγRIIα, CD206 in the right panel). (C) PCA based on MFI of CLR expression on cDC1s of the four groups studied for the two distinct data sets including their respective graph of variables (DCIR, Dectin-1, Clec-9α in the left panels; and DEC-205, Clec-12α, FcγRIIα, CD206 in the right and bottom panels). (D) MFI of DCIR, Dectin-1, Clec-9α, DEC-205, Clec-12α, FcγRIIα and CD206 on cDC1s from the blood of healthy donors (HD, open circles, n=31) and melanoma patients (Pt, filled circles, n=26), and tissue control (open triangles, n=9) and tumor infiltrates of melanoma patients (filled triangles, n=19). Results are expressed as MFI of the CLR amongst CLR-positive cDC1s. Only significant statistics are shown on the graphs. Bars indicate median. *P*-values were calculated using Mann-Withney (dashed lines) and Kruskal-Wallis (full lines) non parametric tests. *P-value ≤ 0.05, **P-value ≤ 0.01, ***P-value ≤ 0.001.

<u>Supplementary figure 6:</u> Modulation of CLR expression on circulating and tumor-infiltrating pDCs in melanoma patients

Circulating and tumor-infiltrating immune cells from melanoma patients and controls were labelled with specific antibodies to depict CD11c$^-$BDCA4$^+$ pDCs amongst alive CD45$^+$Lin$^-$HLA-DR$^+$ cells and their CLR expression was assessed using flow cytometry. (A) Heat map based on the MFI of DCIR, NKp44, ILT7, FcγRIIα, FcεRIα and BDCA2 on pDCs derived from patients' blood (n=16) and tumors (n=13), and controls' blood (n=45) and tissue (n=9). (B) PCA based on MFI of CLR expression on pDCs of the four groups studied (including graph of variables). BDCA2 could not be integrated in this analysis given that it was analyzed in a different dataset from the other CLRs studied for pDCs. (C) MFI of DCIR, NKp44, ILT7, FcγRIIα, FcεRIα and BDCA2 on pDCs from the blood of healthy donors (HD, open circles, n=45) and melanoma patients (Pt, filled circles, n=16), and tissue control (open triangles, n=9) and tumor infiltrates of melanoma patients (filled triangles, n=13). Results are expressed as MFI of the CLR amongst CLR-positive pDCs. Only significant statistics are shown on the graphs. Bars indicate median. *P*-values were calculated using Mann-Withney (dashed lines) and Kruskal-Wallis (full lines) non parametric tests. *P-value ≤ 0.05, ****P-value ≤ 0.0001.

Supplementary figure 7: DCs subsets derived from HD blood exhibit perturbation of their CLR profile after culture with melanoma-derived supernatants

PanDCs (mixture of the three DC subsets (cDC2s, cDC1s, pDCs) were purified from several HD blood and co-cultured with distinct tumor-derived supernatants (50% of total medium) for 2 or 20 hours. Their CLR expression was subsequently assessed using flow cytometry. (A) Basal CLR expression on purified PanDCs at the start of the experiment. (B) Heat map based on the percentage of expression of DCIR, Dectin-1, DEC-205, Clec-12α, CD207 and CD206 on cDC2s; DCIR, Dectin-1, Clec-9α, DEC-205, Clec-12α, FcγRIIα and CD206 on cDC1s; and DCIR, NKp44, ILT7, FcγRIIα, FcεRIα and BDCA2 on pDCs after 2 or 20 hours of culture with (n=6) or without (n=3) distinct tumor supernatants. (C) Expression levels of Dectin-1 and Clec-12α on cDC2s purified from HD blood after 2 or 20 hours of co-culture with tumor supernatants (n=6). (D) Expression levels of DCIR, Clec-9α and FcγRIIα on cDC1s purified from HD blood after 2 or 20 hours of co-culture with tumor supernatants (n=6). (E) Expression levels of DCIR and FcεRIα on pDCs purified from HD blood after 2 or 20 hours of co-culture with tumor cell lines (n=6). (C-E) Results are expressed as percentages of positive cells

within each DC subset. Only statistics with a *P*-value < 0.1 are shown on the graphs. *P*-values were calculated using Wilcoxon matched-pairs signed rank test.

<u>Supplementary figure 8:</u> Higher levels of CD206 by circulating CD206^{+} cDC2s correlate with melanoma progression

Multiple regressions were performed to study the potential link between CLR expression by circulating DCs subsets and disease progression in melanoma patients. (A) PCA based on the MFI of CLR expression by circulating DC subsets derived from patients at different disease stages (n=10 for I-II stages and n=7 for III-IV stages) (including graph of variables). (B) Hazard ratios from comparative OS (from sampling time) of TNM classification and MFI of several CLRs (previously seen modulated in patients' blood when compared to control) by circulating cDC2s of melanoma patients (n= 17). For CLR expression on circulating cDC2s, groups were separated using the median MFI of the CLR by CLR-expressing cDC2s from patients' blood.

<u>Supplementary figure 9:</u> CLR expression profile by circulating and tumor-infiltrating DC subsets correlates with patients' clinical outcome

Survival analyses were performed to study the potential impact of CLR expression by DCs subsets (derived from PBMC and tumor-infiltrating cells from melanoma patients) on clinical outcome. (A) Comparative PFS (from sampling time) and OS (from sampling time) of patients with low or high levels of circulating DCIR^{+} cDC2s (left panel), and CD206^{+} cDC2s (right panels). Groups were separated using the median MFI of DCIR (4953) and CD206 (11614) on circulating DCIR^{+} or CD206^{+} cDC2s (n= 8 to 9 patients/group). (B) Comparative PFS (from sampling time) of patients with low or high levels of circulating Dectin-1^{+} cDC1s. Groups were separated using the median MFI of Dectin-1 (6210) on circulating Dectin-1^{+} cDC1s (n= 7 to 9 patients/group). (C) Comparative PFS (from sampling or diagnostic time) and OS (from diagnostic time) of patients with low or high levels of circulating DCIR^{+} cDC2s and/or Dectin-1^{+} cDC1s (upper left panel), circulating DC-SIGN^{+} cDC2s and/or ILT7^{+} pDCs (upper right panel), DC-SIGN^{+} cDC2s and/or FcγRIIα^{+} pDCs (lower left panel), and DEC-205^{+} cDC2s and/or ILT7^{+} pDCs (lower right panel). Groups were separated using the median MFI of

DCIR (4953) on circulating DCIR^{+} cDC2s and of Dectin-1 (6210) on circulating Dectin-1^{+} cDC1s (n= 6 to 10 patients/group), or the median percentages of circulating DC-SIGN^{+} (2.84%) or DEC-205^{+} (79.85%) cDC2s, and ILT7^{+} (92.33%) or FcγRIIα^{+} (81.60%) pDCs (n= 4 to 12 patients/group). (D) Comparative OS (from sampling time) or PFS and OS (from diagnostic time) of patients with low or high levels of tumor-infiltrating CD206^{+} (left panel) or FcγRIIα^{+} (middle and right panels) cDC1s respectively. Groups were separated using the median MFI of CD206 (3672.5) or FcγRIIα (5099) on tumor-infiltrating CD206^{+} or FcγRIIα^{+} cDC1s respectively (n= 4 patients/group). (A-D) Comparisons using Log-rank test.

2 Supplementary Tables

Suppl Figure 1

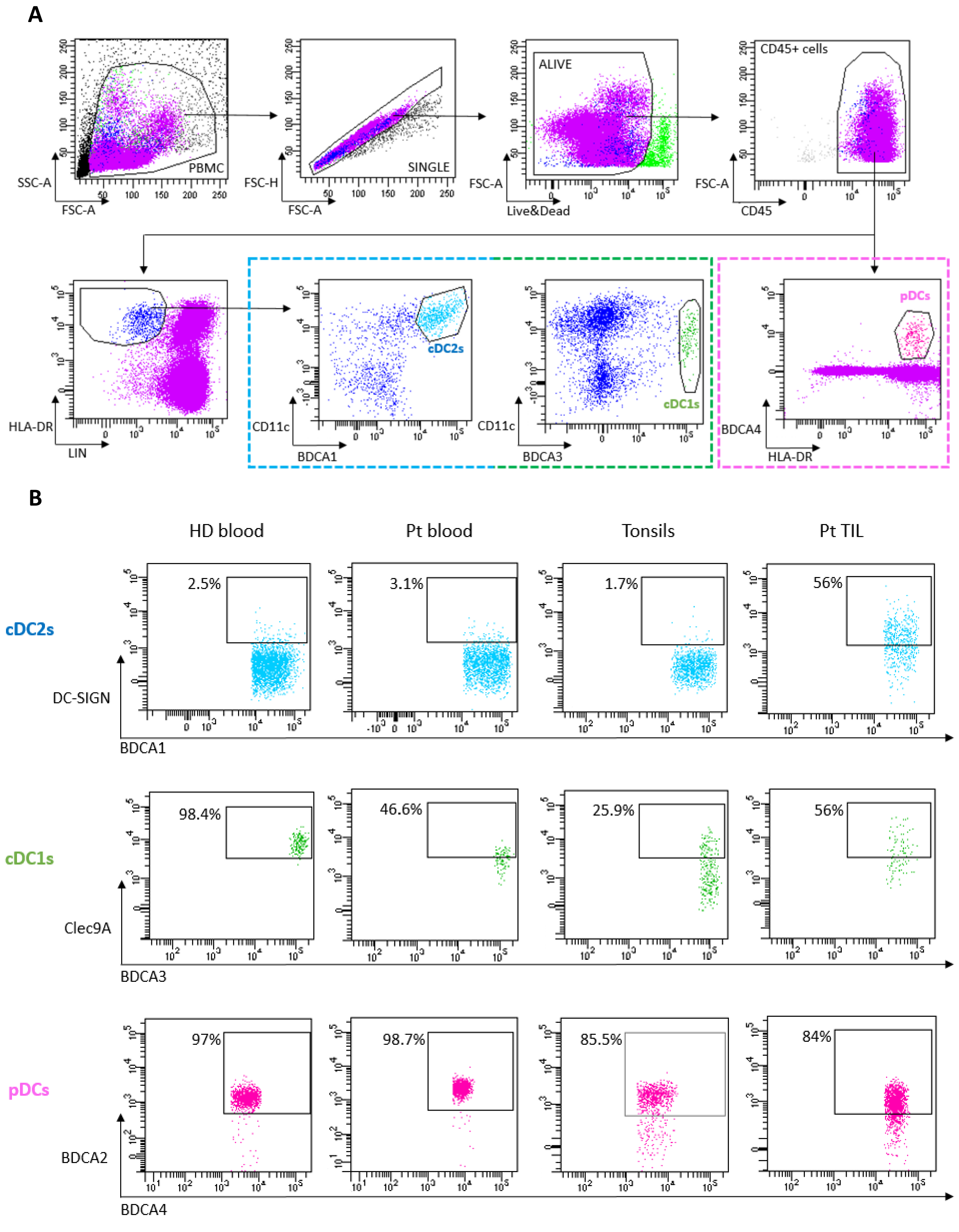

Suppl Figure 2

A

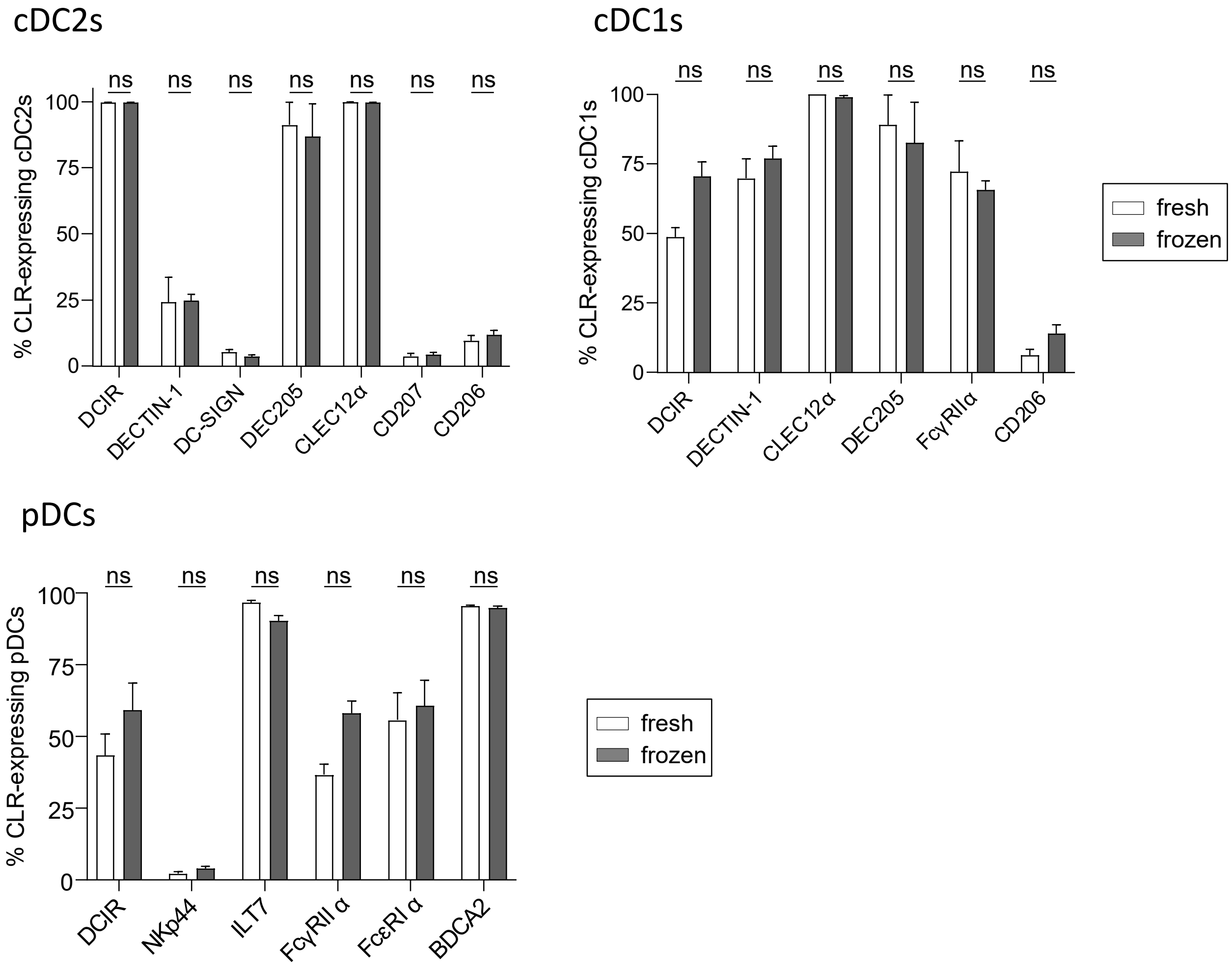

B

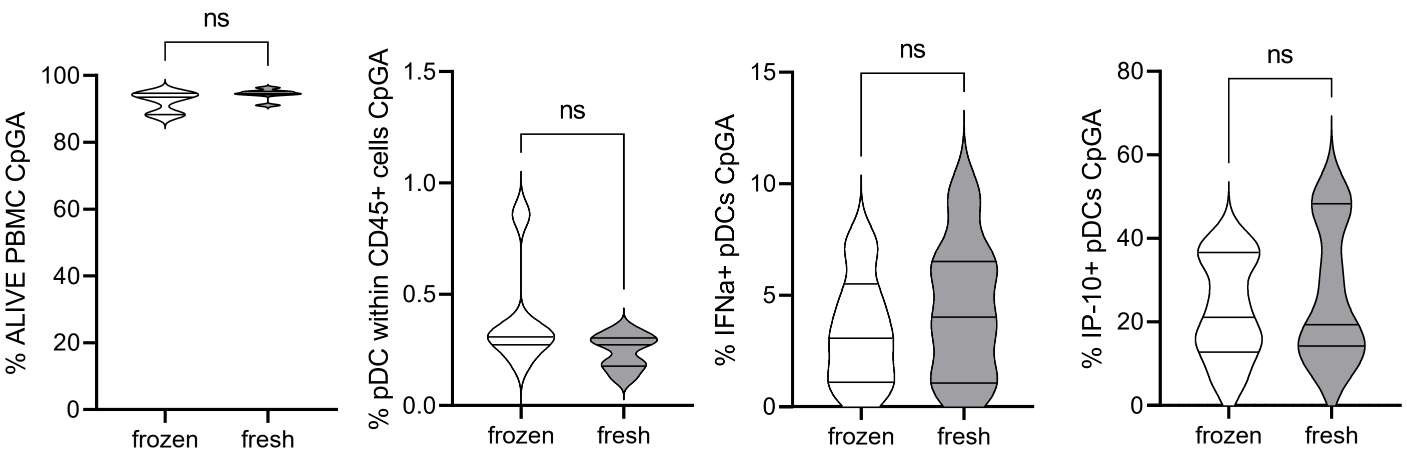

Suppl Figure 3

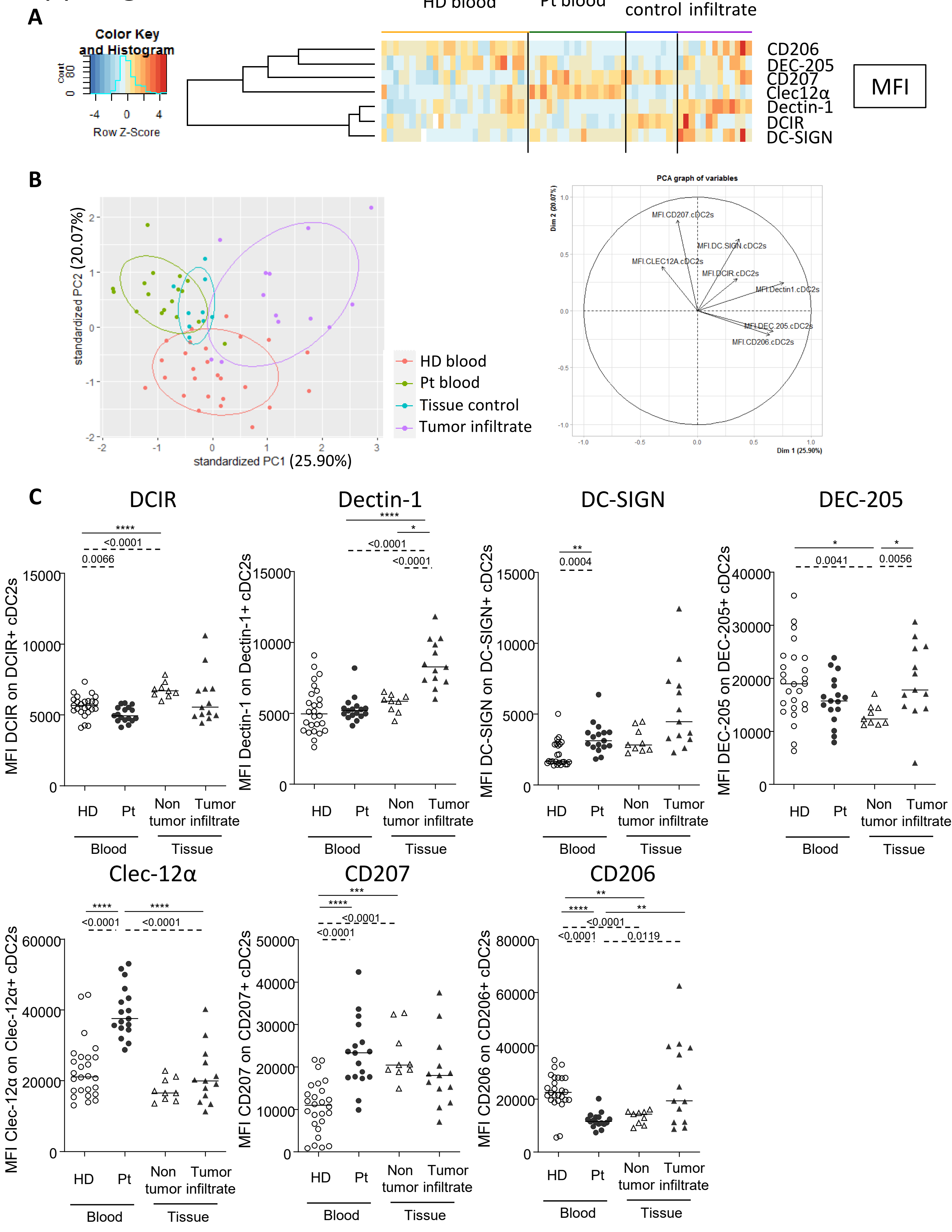

Suppl Figure 4

A

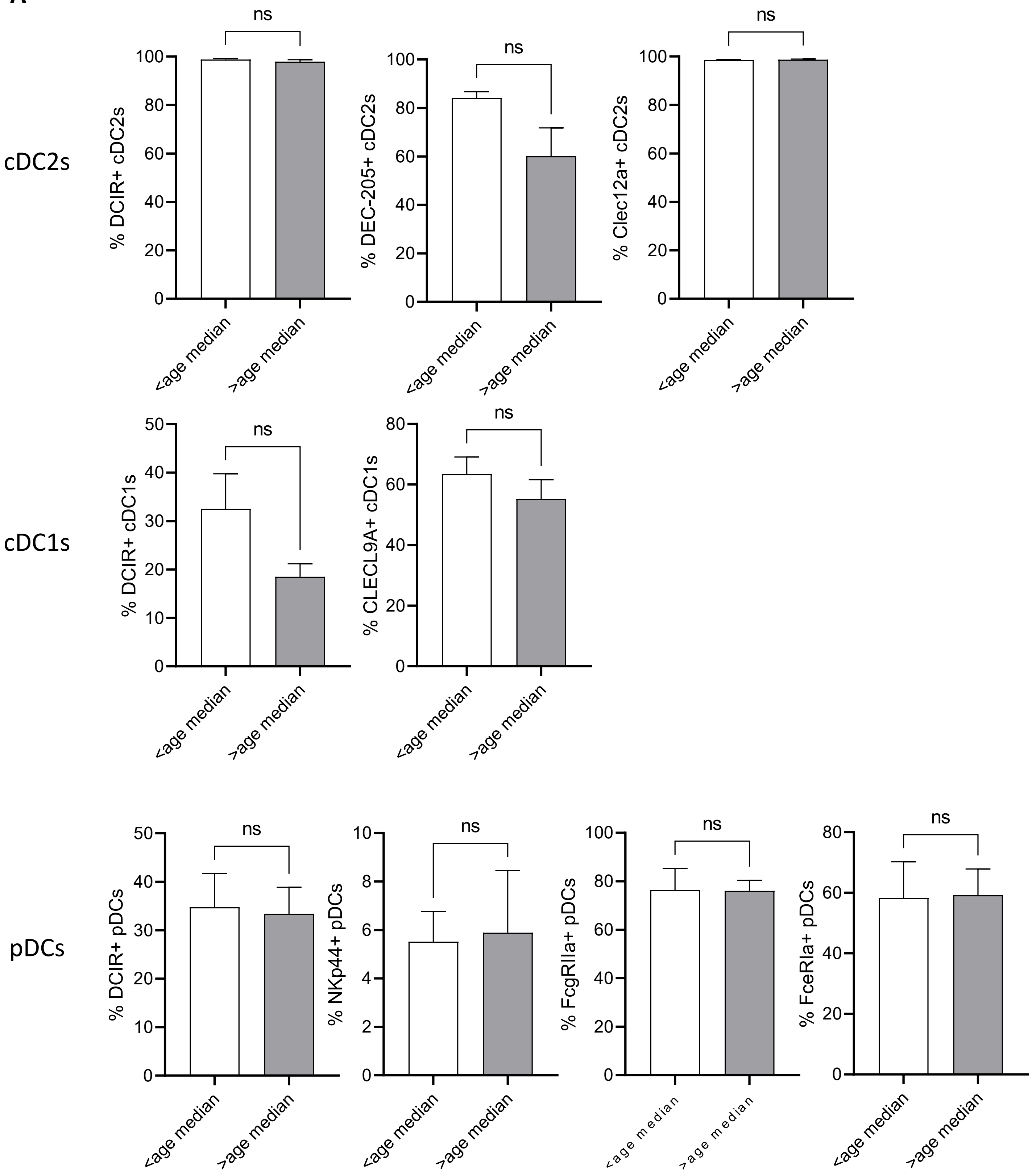

Suppl Figure 4 follow

B

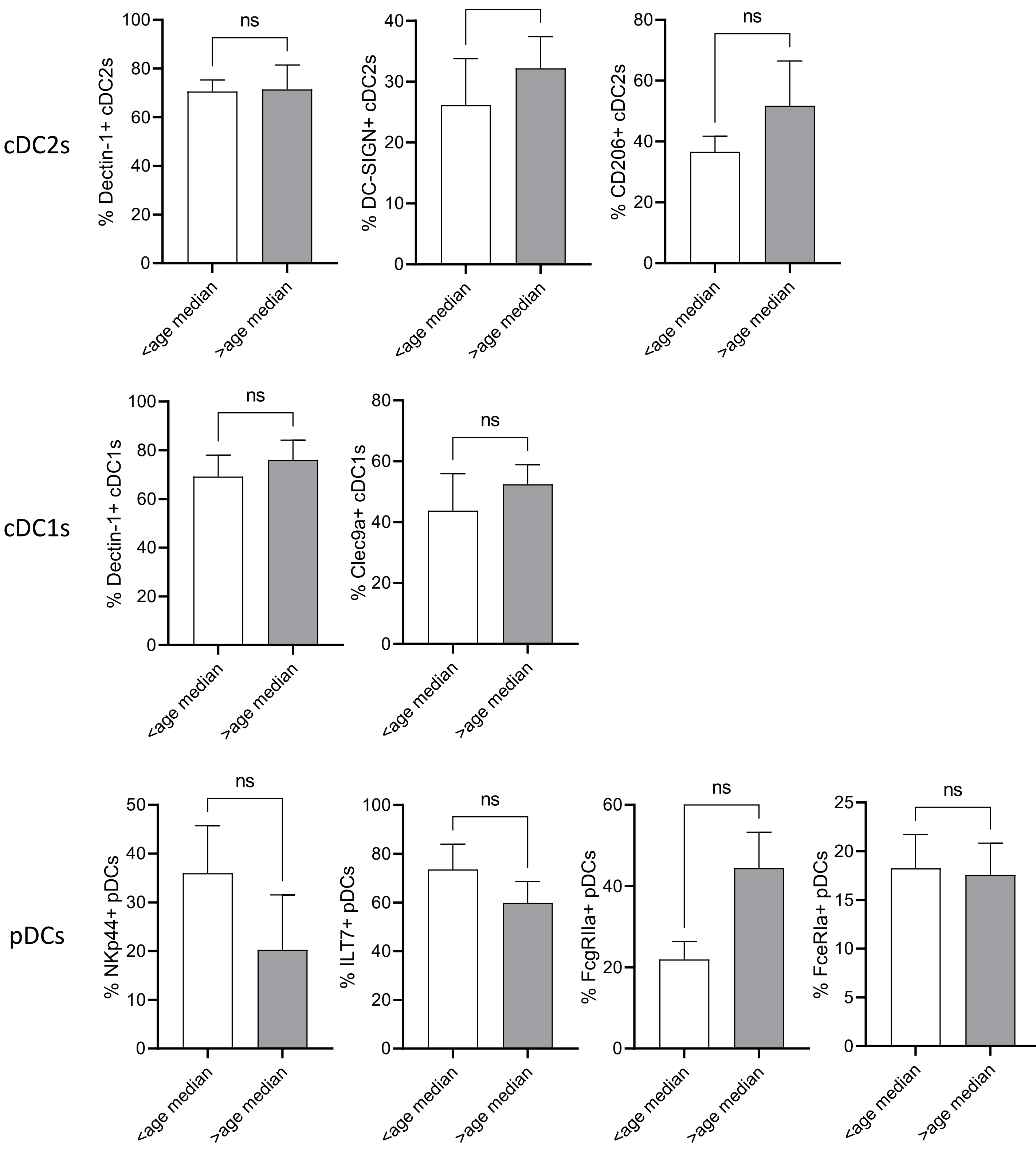

Suppl Figure 5

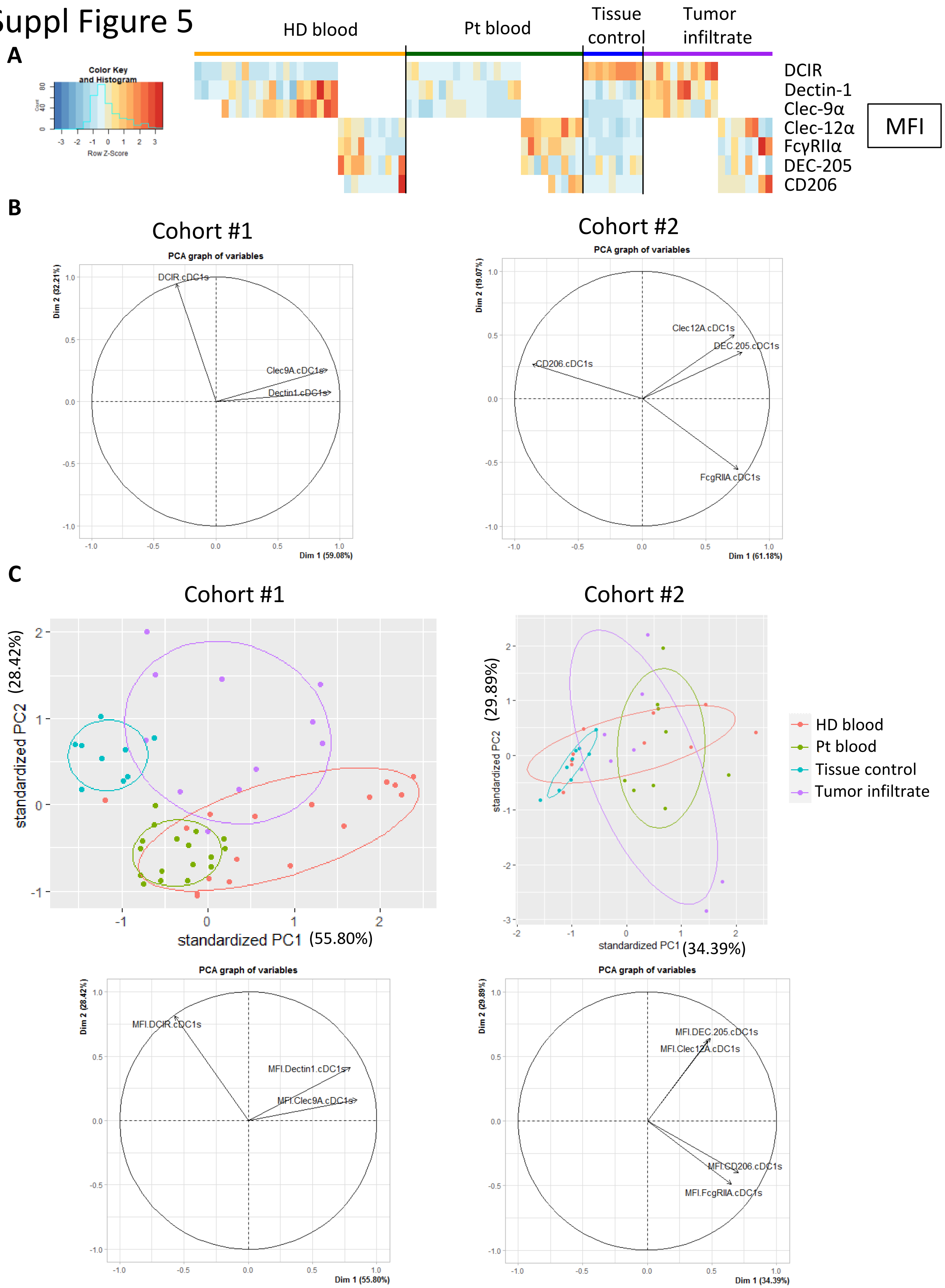

Suppl Figure 5 follow

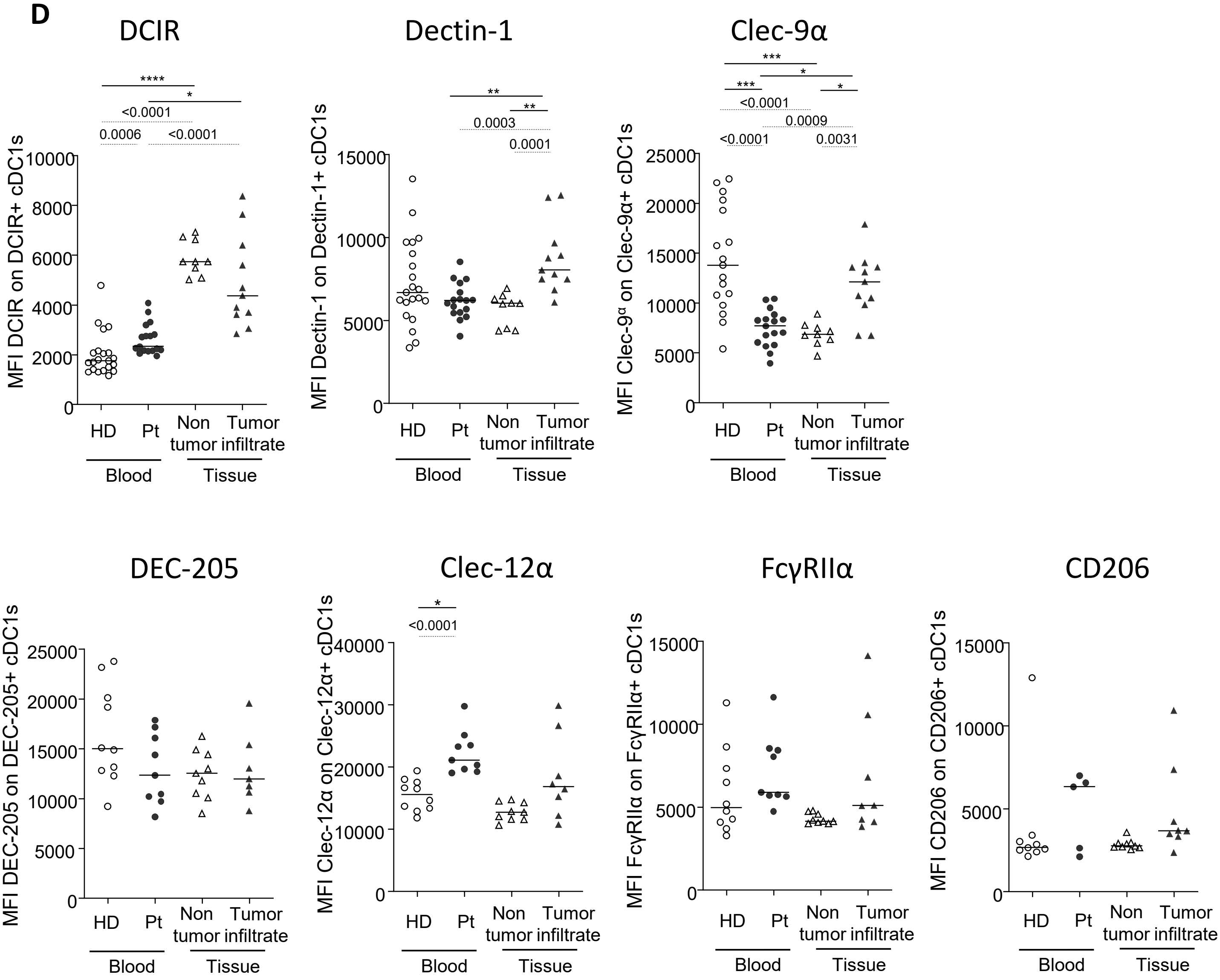

Suppl Figure 6

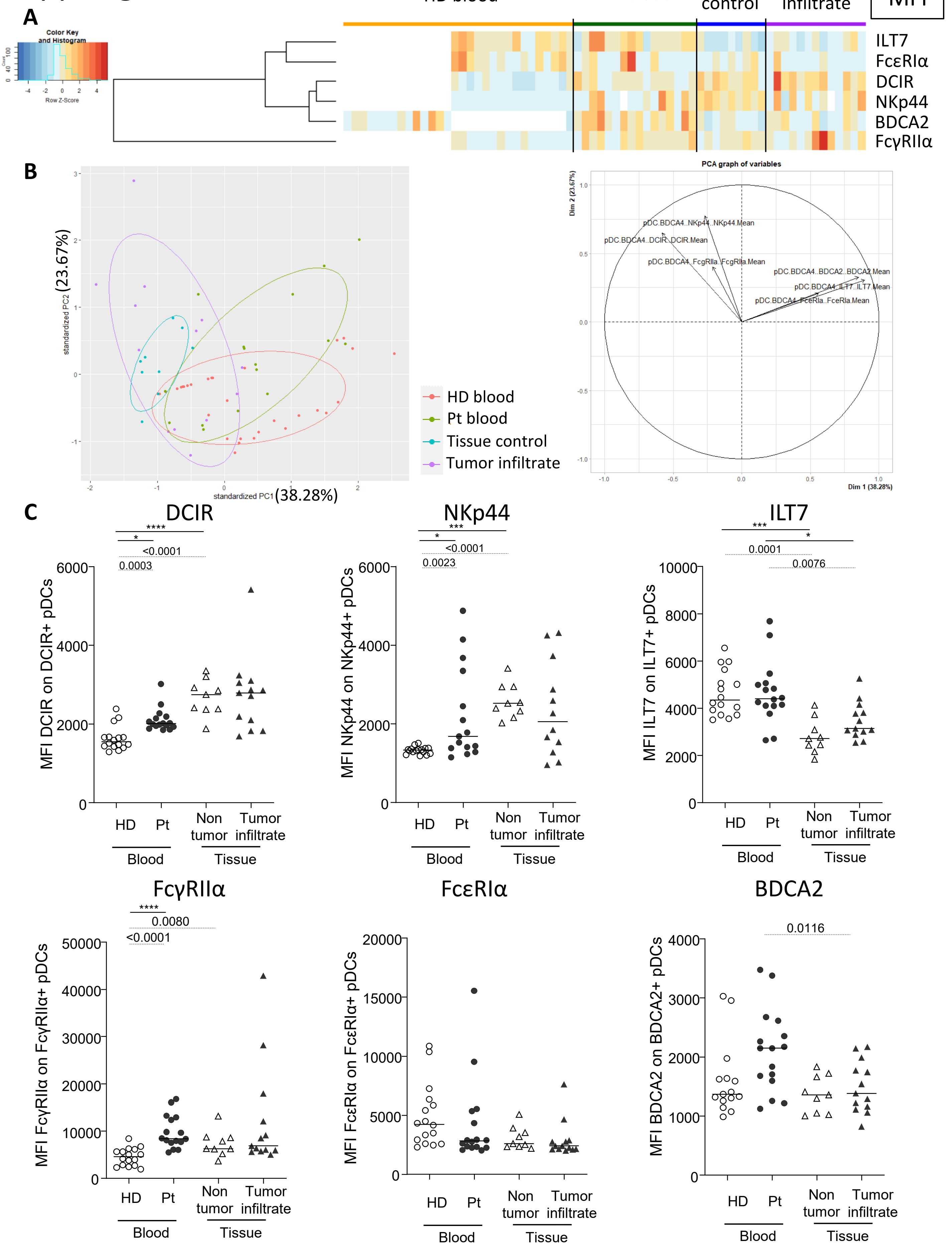

Suppl Figure 7

A

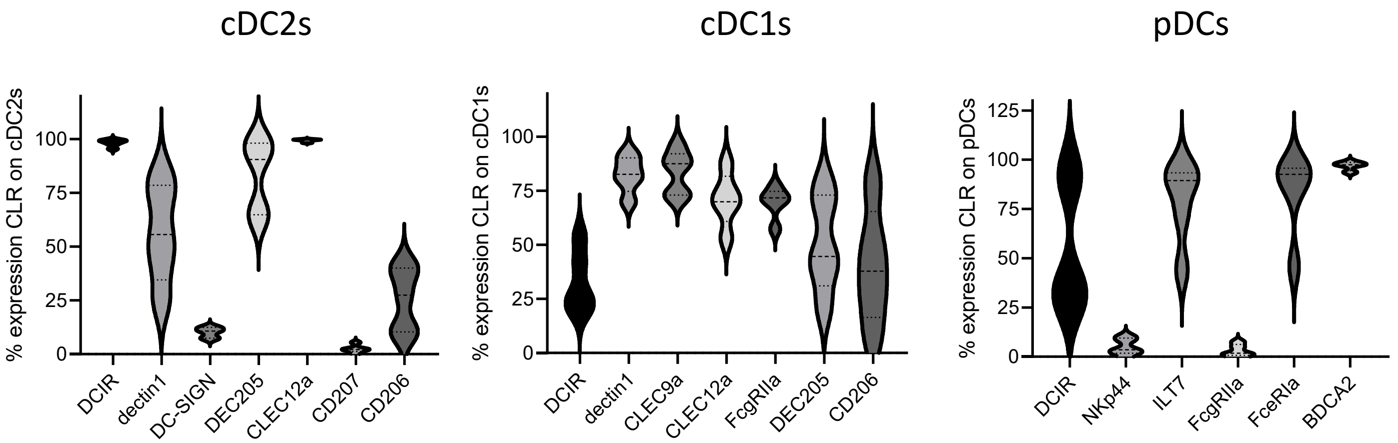

B

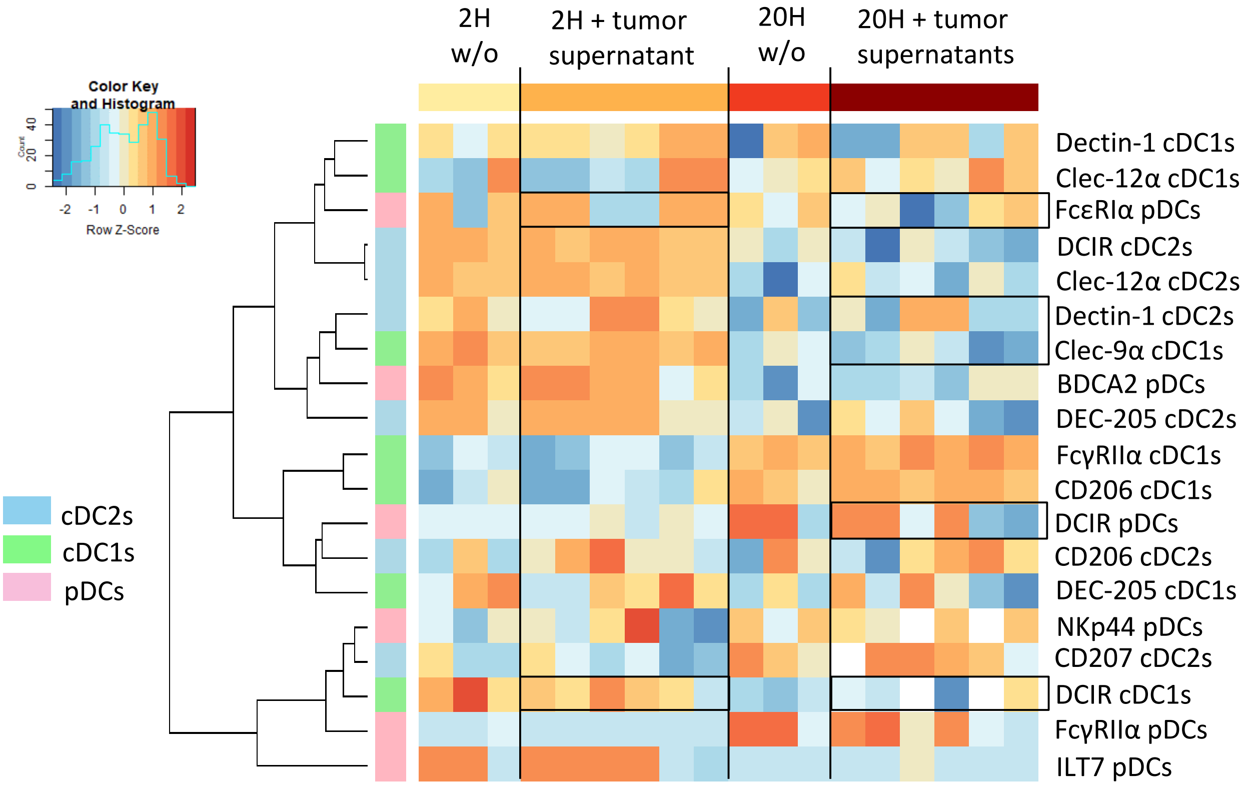

Suppl Figure 7 follow

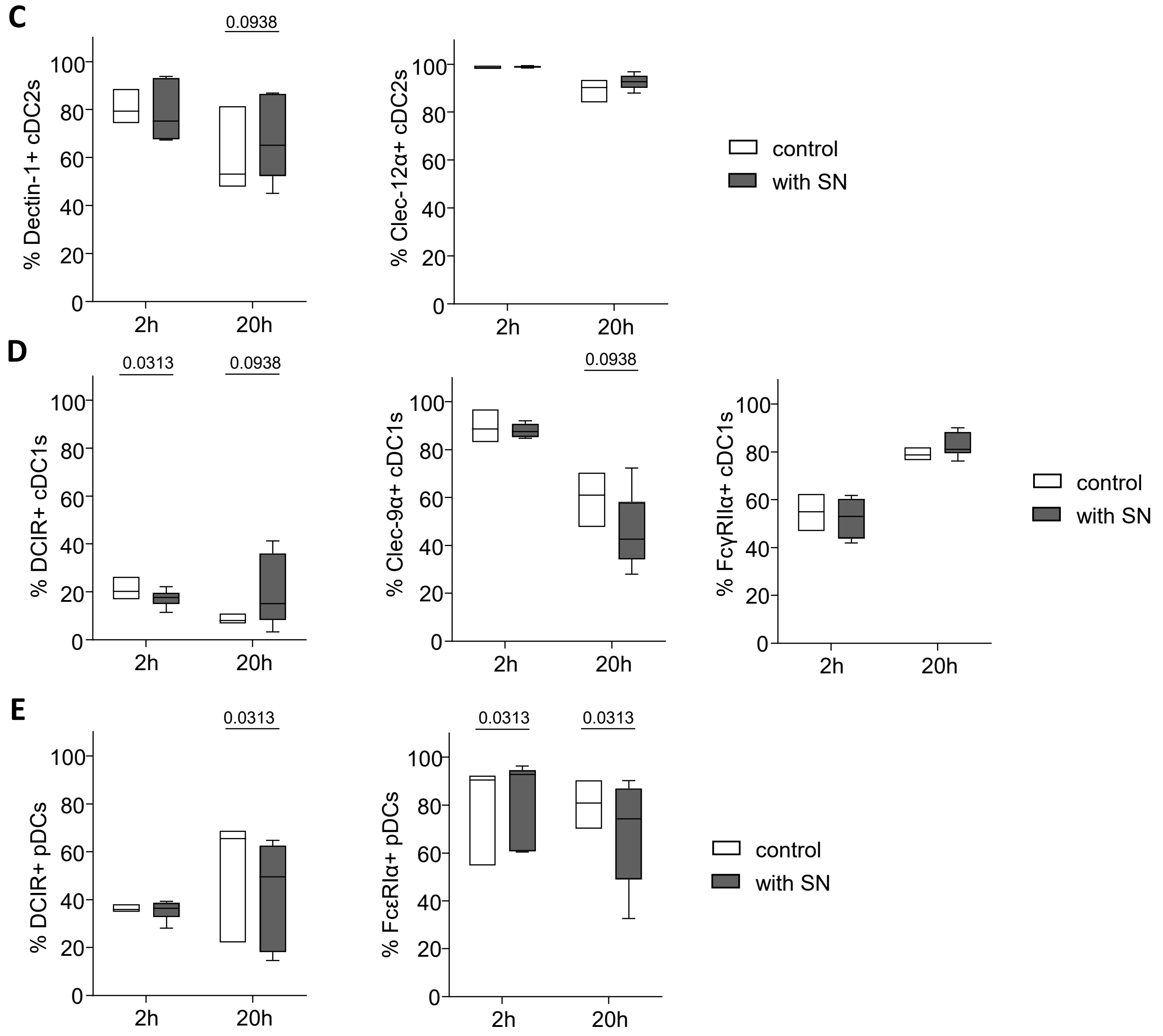

Suppl Figure 8

A Pt blood

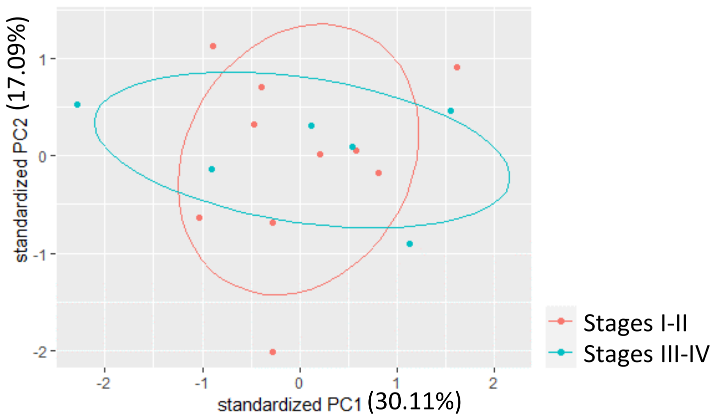

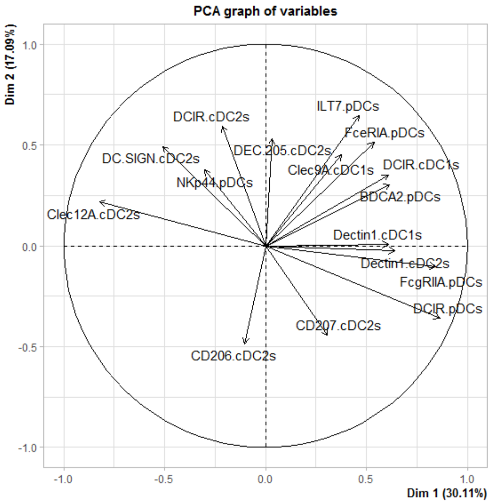

B Pt blood

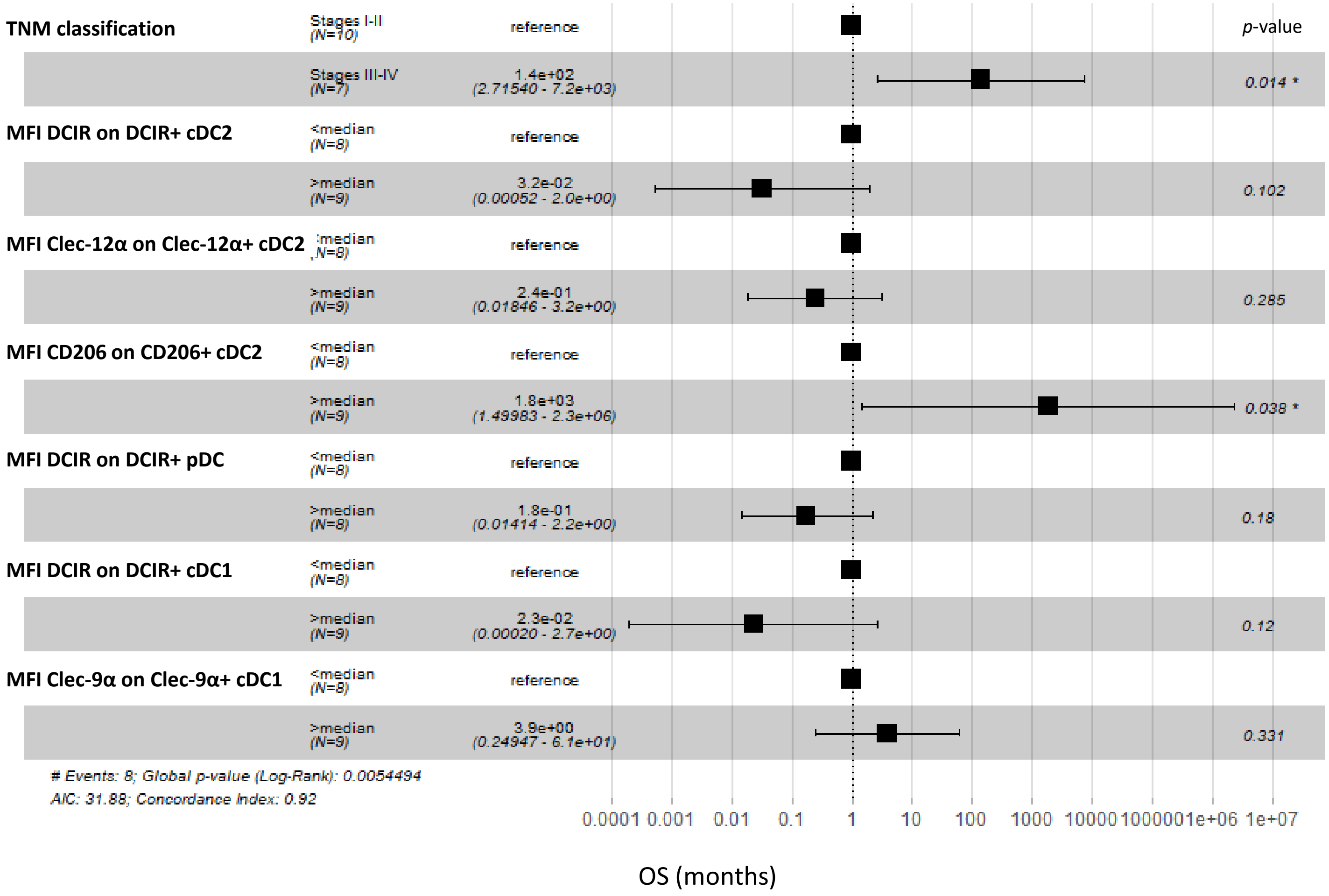

Suppl Figure 9

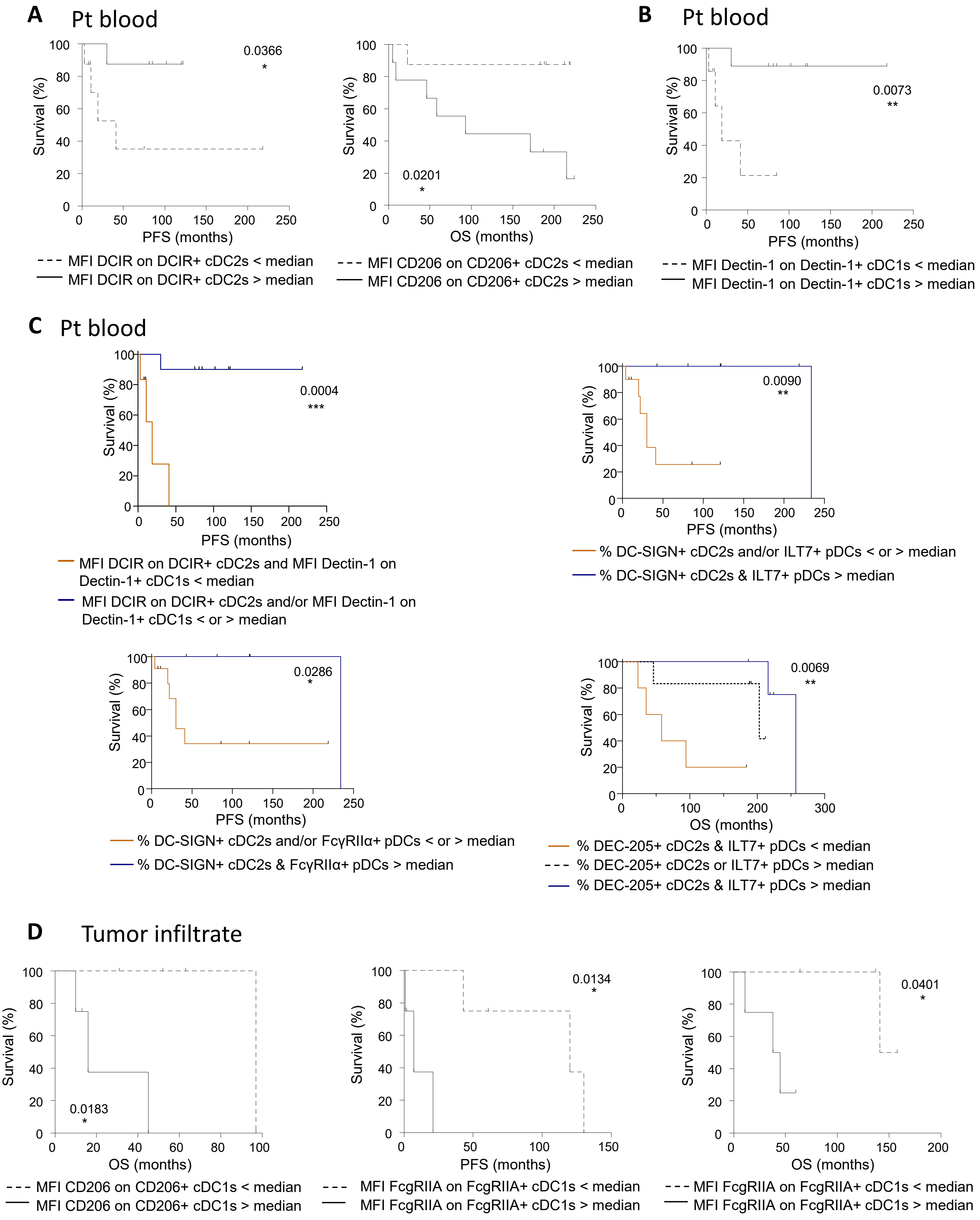

Supplementary table 1 : Clinical features of patients from whom blood samples were used

Patient clinical features									From diagnosis time		From sampling time	
#	sample type	sex	age	Breslow (mm)	Clark	Ulcer	treatment before sampling	TNM classification* (at sampling time)	PFS	OS	PFS	OS
1	PBMC	F	38	3	IV	yes	no	IIIc (T3b N1a M0)	41	46	41	46
2	PBMC	F	48	1.6	IV	yes	no	IIa (T2b N0 M0)	>122	>224	>122	>224
3	PBMC	M	62	1.2	III	ND	no	Ib or IIa (T2 N0 MO)	>121	216	>120	215
4	PBMC	M	73	0.71 / 0.95	III / ND	no /no	surgery	Ib (T1b N0 M0)	>43	203	>75	171
5	PBMC	M	66	1.9	IV	yes	ND	IIa (T2b N0 M0)	>219	>219	>218	>218
6	PBMC	F	41	2.8	III	no	surgery; chemotherapy: IFNα	IV (T3a N1b M1a)	4	23	3	9
7	PBMC	M	48	1.35	III	no	no	IIIa (T2a N2a M0)	>103	>220	>102	>219
8	PBMC	M	89	4 / 2	ND / IV	ND	surgery	IV (T3 Nx M1a)	234	257	11	23
9	PBMC	M	35	1	IV	no	no	IIIa (T1a N1a M0)	>121	>189	>120	>188
10	PBMC	F	42	1.05	IV	no	surgery	IIIc (T2a N2c M0)	22	>212	>85	>212
11	PBMC	F	80	4.1	IV	yes	no	IIc (T4b N0 M0)	20	94	19	93
12	PBMC	F	45	0.54	III	no	no	Ia (T1a N0 M0)	>8	>188	>8	>188
13	PBMC	F	49	0.4 / 1.62	III / III	no	surgery	IV (T2a N1 M1c)	30	35		5
14	PBMC	F	64	3	IV	no	no	IIa (T3a N0 M0)	>86	>191	>85	>190
15	PBMC	F	72	1.1	IV	no	no	Ib (T2a N0 M0)	>11	>184	>10	>183
16	PBMC	F	61	2.6	IV	yes	no	IIb (T3b N0 M0)	30	58	30	58
17	PBMC	F	45	1.5	IV	no	no	Ib (T2a N0 M0)	>81	>187	>81	>187
18	PBMC	F	35	3	II	no	no	IIa (T3a N0 M0)	>109	>214	>109	>214
19	PBMC	F	81	3.1	IV	yes	no	IIIc (T3b N1a M0)	>42	154	>41	153
20	PBMC	M	41	2	IV	ND	no	Ib or IIa (T2 N0 M0)	>108	>218	>92	>212
21	PBMC	M	50	6	IV	ND	no	IIb or IIc (T4 N0 M0)	>48	117	>47	116
22	PBMC	F	46	1.02 / 0.28	III/ II	no	no	Ib (T2a N0 M0)	>208	>208	>208	>208
23	PBMC	F	63	1.3	IV	no	no	Ib (T2a N0 M0)	>192	>192	>191	>191
24	PBMC	F	34	1.2	III	ND	no	Ib or IIa (T2 N0 M0)	>85	>200	>84	>199
25	PBMC	M	67	1.8	V	yes	no	IIa (T2b N0 M0)	>95	>193	>94	>192
26	PBMC	M	77	4.2	III	yes	no	IIc (T4b N0 M0)	>73	>214	>72	>213

ND: not determined

* 8th edition

Supplementary table 2: Clinical features of patients from whom tumor samples were used

Patient clinical features									From diagnosis time		From sampling time	
#	sample type	sex	age	Breslow (mm)	Clark	Ulcer	treatment before sampling	TNM classification* (at sampling time)	PFS	OS	PFS	OS
27	primary tumor or sub-cutaneous	F	80	ND	ND	ND	no	IIIc (TxN3M0)		42		42
28	lymph node metastasis	M	26	2.64	IV	no	surgery; IFNα ; radiotherapy	IV (T3aN3M1a)	14	84	12	31
29	ND	F	51	0.8	III	no	no	ND	50	86		36
30	lymph node metastasis	F	69	0.78	III	no	surgery; chemotherapy; vaccination (DC MEL 201)	IV (T1a Nx M1a)	44	107	6	19
31	lymph node metastasis	M	25	ND	ND	ND	no	ND		31		30
32	primary tumor or lymph node metastasis	M	62	1.4	III	yes	no	IIIb (T2b N1b M0)	17	27	17	27
33	ND	F	72	3.09	IV	yes	no	ND	4	6		2
34	ND	M	39	9	IV	yes	surgery	ND	22	57	22	57
35	ND	F	68	6	V	no	no	ND	94	>288	>90	>195
36	sub-cutaneous metastasis	F	67	1.3	III	no	surgery	IV (T2a N0 M1a)	37	43	3	3
37	primary tumor	M	66	14	V	ND	no	IIIb or IIIc (T4 N2c M0)	13	23	2	11
38	ND	M	46	1.4	IV	no	no	ND	160	213	36	58
39	primary tumor	F	42	0.9	III	no	no	Ia (T1a N0 M0)	>193	>199	>192	>198
40	lymph node metastasis	M	56	8	IV	no	IFNα	IV (T1a N3 M1c)	113	117		4
41	lymph node metastasis	F	72	2.5	IV	no	surgery	IIIb (T3a N2b M0)	120	>137	>13	>13
42	cutaneous metastasis	F	70	ND	ND	ND	chemotherapy ; radiotherapy	IV (Tx Nx M1a)	>61	>64	>60	>63
43	ND	M	75	3	IV	yes	surgery	IIIc (T3b N3 M0)	7	>60	>46	>52
44	ND	F	46	1.4	III	no	no	IV (T2a N1b M1d)	1	11	8	10
45	ND	F	50	4	IV	ND	surgery	IIIc (T4 N1b M0)	130	>158	>27	>31
46	lymph node metastasis	F	54	1.2	III	ND	no	IIIc (T2 N3 M0)	43	141		97
47	lymph node metastasis	F	47	1.75	ND	no	no	IIIa (T2a N1a M0)	21	38	9	16
48	ND	F	89	7	ND	yes	no	IIc (T4b N0 M0)	>2	45	>2	45

ND: not determined
* 8th edition

Supplementary Table 3: Panel of lectins used for GLYcoPROFILES (lectin array) and their glycan structures specificity

Short name	Common name	Glycan structures specificities
BC2L-A	***B**urkholderia* ***C**enocepacia* ***L**ectin* ***A***	Dimanoside : Man(α-1,3)Man > Man(α-1,6)Man > Man(α-1,2)Man
ConA	***Con**canavalin* ***A**gglutinin*	αMan > αGlc
GNA	***G**alanthus* ***N**ivalis* ***A**gglutinin*	Terminal αMan, Man(α-1,3)Man
HPA	***H**elix* ***P**omatia* ***A**gglutinin*	Terminal αGalNAc
PNA	***P**eanut* ***A**gglutinin*	Lactose, Galβ(1,3)GalNAc (T- antigen)
PSA	***P**isum* ***S**ativum* ***A**gglutinin*	αMan/αGlc > αGlcNAc, α6 fucosylation of the N-linked GlcNAc promotes binding.
RPLGal4	***R**ecombinant* ***P**rokaryotic* ***L**ectin* ***Gal**actose* ***4***	Terminal βGal, LacNAc and Lewis x
RPLαMan	***R**ecombinant* ***P**rokaryotic* ***L**ectin* ***αMan**nose*	Fuc/Man: Lewis a, Lewis x and terminal αMan
WGA	***W**heat* ***G**erm* ***A**gglutinin*	GlcNAc; GlcNAcβ4 oligomers, core of Asn linked oligasacchide; Neu5Ac

Supplementary Table 4: Correlation between CLR expression of DC subsets and the glyco-code of melanoma cells in patients (Spearman correlation)

Spearman correlation (*r*, *P*-value)	% ConA		% PSA		% GNA		% BCLA-2		% RPL-αMan		% WGA	
	r	*P*-value	*r*	*P*-value	*r*	*P*-value	*r*	*P*-value	*r*	*P*-value	*r*	*P*-value
% DCIR+ cDC2s	0.7	*0.23*	0.7	*0.23*	0.1	*0.95*	0.2	*0.78*	0.1	*0.95*	1	*0.02*
% Dectin-1+ cDC2s	0.1	*0.95*	0.4	*0.52*	-0.5	*0.45*	-0.1	*0.95*	-0.5	*0.45*	0.1	*0.95*
% DC-SIGN+ cDC2s	-0.7	*0.23*	-0.3	*0.68*	-0.9	*0.08*	-0.7	*0.23*	-0.9	*0.08*	-0.3	*0.68*
% CD206+ cDC2s	0.2	*0.78*	0.3	*0.68*	-0.6	*0.35*	-0.3	*0.68*	-0.6	*0.35*	0.3	*0.68*
% DCIR+ pDCs	0.5	*0.45*	0.7	*0.23*	-0.1	*0.95*	0.3	*0.68*	-0.1	*0.95*	0.3	*0.68*
% DCIR+ cDC1ss	-0.8	*0.33*	-0.6	*0.42*	-0.4	*0.75*	-0.4	*0.75*	-0.4	*0.75*	-0.6	*0.42*
% Dectin-1+ cDC1s	0.8	*0.33*	1	*0.08*	0.4	*0.75*	0.4	*0.75*	0.4	*0.75*	1	*0.08*

Supplementary table 5: Impact of the CLR expression of circulating or tumor-infiltrating DC subsets on clinical outcome (Cox regression analysis)

COX regression (HR, *P*-values)	Patient blood								Patient tumor infiltrate							
	PFS diagnosis		OS diagnosis		PFS sampling		OS sampling		PFS diagnosis		OS diagnosis		PFS sampling		OS sampling	
	HR	*P*-value	HR	*P*-value	HR	*P*-value	HR	*P*-value	HR	*P*-value	HR	*P*-value	HR	*P*-value	HR	*P*-value
% DCIR+ cDC2s	0.803	*0.347*	0.930	*0.724*	0.698	*0.074*	0.811	*0.202*	1.071	*0.642*	1.120	*0.382*	1.070	*0.673*	1.071	*0.569*
% Dectin-1+ cDC2s	0.929	*0.106*	0.958	*0.246*	0.999	*0.983*	0.997	*0.923*	1.044	*0.150*	1.031	*0.305*	1.031	*0.297*	1.031	*0.238*
% DC-SIGN+ cDC2s	0.957	*0.658*	1.091	*0.210*	1.045	*0.588*	1.095	*0.135*	0.987	*0.452*	0.991	*0.622*	0.981	*0.444*	0.987	*0.482*
% DEC-205+ cDC2s	0.984	*0.229*	0.985	*0.235*	0.984	*0.530*	0.989	*0.347*	1.031	*0.076*	1.020	*0.200*	1.020	*0.341*	1.013	*0.308*
% Clec-12α+ cDC2s	0.923	*0.894*	1.291	*0.660*	0.626	*0.546*	0.660	*0.457*	1.030	*0.350*	0.996	*0.888*	1.018	*0.677*	0.990	*0.746*
% CD207+ cDC2s	1.368	*0.389*	1.109	*0.739*	0.801	*0.646*	0.864	*0.652*	0.926	*0.039*	0.969	*0.211*	0.961	*0.276*	0.979	*0.382*
% CD206+ cDC2s	1.027	*0.685*	1.116	*0.103*	1.053	*0.441*	1.060	*0.310*	1.007	*0.553*	0.992	*0.526*	0.988	*0.442*	0.996	*0.723*
MFI DCIR+ cDC2s	0.999	*0.157*	0.999	*0.215*	0.998	*0.057*	0.999	*0.182*	1.000	*0.060*	1.000	*0.202*	1.000	*0.350*	1.000	*0.437*
MFI Dectin-1+ cDC2s	0.997	*0.058*	1.000	*0.796*	0.998	*0.116*	1.000	*0.860*	1.000	*0.297*	1.000	*0.819*	1.000	*0.488*	1.000	*0.620*
MFI DC-SIGN+ cDC2s	1.000	*0.170*	1.000	*0.387*	1.000	*0.952*	1.000	*0.548*	1.000	*0.200*	1.000	*0.356*	1.000	*0.375*	1.000	*0.209*
MFI DEC-205+ cDC2s	1.000	*0.138*	1.000	*0.651*	1.000	*0.140*	1.000	*0.721*	1.000	*0.960*	1.000	*0.859*	1.000	*0.414*	1.000	*0.923*
MFI Clec-12α+ cDC2s	1.000	*0.838*	1.000	*0.377*	1.000	*0.651*	1.000	*0.677*	1.000	*0.345*	1.000	*0.516*	1.000	*0.902*	1.000	*0.633*
MFI CD207+ cDC2s	1.000	*0.678*	1.000	*0.457*	1.000	*0.598*	1.000	*0.269*	1.000	*0.116*	1.000	*0.441*	1.000	*0.199*	1.000	*0.311*
MFI CD206+ cDC2s	1.000	*0.053*	1.000	*0.045*	1.000	*0.185*	1.000	*0.142*	1.000	*0.036*	1.000	*0.055*	1.000	*0.083*	1.000	*0.115*
% DCIR+ cDC1s	0.944	*0.210*	0.944	*0.226*	0.960	*0.361*	0.970	*0.350*	1.000	*0.974*	1.002	*0.904*	1.011	*0.540*	1.011	*0.486*
% Dectin-1+ cDC1s	0.945	*0.222*	0.925	*0.081*	0.958	*0.367*	0.964	*0.342*	1.022	*0.239*	0.999	*0.957*	1.000	*0.983*	0.996	*0.789*
% Clec-9α+ cDC1s	0.987	*0.586*	0.966	*0.146*	0.985	*0.548*	0.982	*0.386*	1.001	*0.948*	0.991	*0.599*	0.986	*0.658*	0.992	*0.680*
% Clec-12α+ cDC1s	NA	*NA*	1.084	*0.871*	NA	*NA*	1.084	*0.871*	0.921	*0.252*	0.819	*0.066*	0.877	*0.176*	0.832	*0.093*
% FcγRIIα+ cDC1s	NA	*NA*	0.997	*0.956*	NA	*NA*	0.997	*0.956*	0.997	*0.901*	0.991	*0.801*	0.991	*0.908*	0.966	*0.492*
% DEC-205+ cDC1s	NA	*NA*	0.961	*0.171*	NA	*NA*	0.961	*0.171*	0.979	*0.253*	0.956	*0.113*	0.953	*0.315*	0.964	*0.207*
% CD206+ cDC1s	NA	*NA*	1.6e-08	*0.999*	NA	*NA*	1.6e-08	*0.999*	1.162	*0.086*	1.105	*0.167*	1.120	*0.250*	1.106	*0.231*
MFI DCIR+ cDC1s	1.000	*0.473*	1.000	*0.437*	1.001	*0.441*	1.000	*0.782*	1.000	*0.264*	1.000	*0.487*	1.000	*0.345*	1.000	*0.656*
MFI Dectin-1+ cDC1s	0.999	*0.054*	0.999	*0.280*	0.999	*0.071*	1.000	*0.290*	1.000	*0.316*	1.000	*0.759*	1.000	*0.928*	1.000	*0.755*
MFI Clec-9α+ cDC1s	1.000	*0.985*	1.000	*0.131*	1.000	*0.480*	1.000	*0.487*	1.000	*0.804*	1.000	*0.948*	1.000	*0.244*	1.000	*0.855*
MFI Clec-12α+ cDC1s	NA	*NA*	1.000	*0.925*	NA	*NA*	1.000	*0.925*	1.000	*0.450*	1.000	*0.227*	1.000	*0.331*	1.000	*0.264*
MFI FcγRIIα+ cDC1s	NA	*NA*	1.000	*0.893*	NA	*NA*	1.000	*0.893*	1.000	*0.308*	1.000	*0.135*	1.000	*0.291*	1.000	*0.240*
MFI DEC-205+ cDC1s	NA	*NA*	1.000	*0.634*	NA	*NA*	1.000	*0.634*	1.000	*0.311*	1.000	*0.223*	1.000	*0.292*	1.000	*0.340*
MFI CD206+ cDC1s	NA	*NA*	NA	*NA*	NA	*NA*	NA	*NA*	1.000	*0.335*	1.000	*0.075*	1.000	*0.232*	1.000	*0.114*
% DCIR pDCs	0.992	*0.755*	0.977	*0.355*	1.000	*0.990*	0.996	*0.857*	1.006	*0.766*	0.998	*0.923*	0.986	*0.559*	0.996	*0.789*
% NKp44+ pDCs	0.971	*0.703*	1.029	*0.548*	0.979	*0.794*	1.009	*0.864*	1.001	*0.926*	0.997	*0.798*	0.998	*0.907*	1.003	*0.861*
% ILT7+ pDCs	0.866	*0.006*	0.882	*0.012*	0.889	*0.038*	0.913	*0.054*	0.979	*0.115*	0.991	*0.480*	1.003	*0.844*	1.003	*0.779*
% FcγRIIα+ pDCs	0.963	*0.098*	0.967	*0.114*	0.961	*0.116*	0.979	*0.301*	1.030	*0.222*	1.010	*0.655*	1.024	*0.495*	1.017	*0.481*
% FcεRIα+ pDCs	0.967	*0.021*	0.975	*0.096*	0.981	*0.253*	0.990	*0.468*	1.020	*0.661*	0.997	*0.941*	0.946	*0.448*	0.996	*0.923*
% BDCA2+ pDCs	0.815	*0.146*	0.705	*0.026*	0.789	*0.139*	0.830	*0.202*	0.981	*0.219*	0.993	*0.631*	1.010	*0.611*	1.007	*0.588*
MFI DCIR pDCs	1.000	*0.911*	1.001	*0.215*	1.000	*0.693*	1.001	*0.358*	1.001	*0.102*	1.000	*0.504*	1.001	*0.194*	1.000	*0.400*
MFI NKp44+ pDCs	1.000	*0.938*	1.001	*0.075*	1.000	*0.762*	1.001	*0.039*	1.000	*0.896*	1.000	*0.292*	1.000	*0.585*	1.000	*0.752*
MFI ILT7+ pDCs	0.999	*0.053*	1.000	*0.626*	0.999	*0.084*	1.000	*0.818*	0.999	*0.139*	1.000	*0.321*	0.999	*0.350*	1.000	*0.462*
MFI FcγRIIα+ pDCs	1.000	*0.148*	1.000	*0.079*	1.000	*0.180*	1.000	*0.229*	1.000	*0.144*	1.000	*0.641*	1.000	*0.821*	1.000	*0.907*
MFI FcεRIα+ pDCs	0.998	*0.143*	1.000	*0.214*	1.000	*0.757*	1.000	*0.659*	1.000	*0.112*	1.000	*0.851*	1.000	*0.503*	1.000	*0.924*
MFI BDCA2+ pDCs	0.999	*0.138*	1.000	*0.830*	0.999	*0.403*	1.000	*0.736*	0.999	*0.406*	1.000	*0.983*	1.000	*0.641*	1.000	*0.993*

Supplementary table 6: Impact of the CLR expression of circulating or tumor-infiltrating DC subsets (% and MFI) on clinical outcome (Log rank test analysis).

Log-rank (*P*-values)	Patient blood				Patient tumor infiltrate			
	PFS diagnosis	OS diagnosis	PFS sampling	OS sampling	PFS diagnosis	OS diagnosis	PFS sampling	OS sampling
% DCIR+ cDC2s	0.439	0.789	0.942	0.363	0.705	0.720	0.673	0.698
% Dectin-1+ cDC2s	0.159	0.561	0.601	0.917	0.186	0.420	0.085	0.285
% DC-SIGN+ cDC2s	0.040	0.317	0.501	0.646	0.984	0.686	0.544	0.587
% DEC-205+ cDC2s	0.075	0.018	0.155	0.121	0.181	0.125	0.488	0.454
% Clec-12α+ cDC2s	0.780	0.543	0.942	0.842	0.441	0.794	0.539	0.891
% CD207+ cDC2s	0.798	0.740	0.549	0.788	0.185	0.729	0.618	0.987
% CD206+ cDC2s	0.356	0.105	0.539	0.364	0.904	0.738	0.706	0.854
MFI DCIR+ cDC2s	0.490	0.490	0.037	0.211	0.705	0.720	0.673	0.698
MFI Dectin-1+ cDC2s	0.536	0.553	0.349	0.926	0.562	0.833	0.944	0.832
MFI DC-SIGN+ cDC2s	0.474	0.630	0.281	0.996	0.895	0.630	0.596	0.288
MFI DEC-205+ cDC2s	0.639	0.837	0.163	0.423	0.862	0.974	0.918	0.721
MFI Clec-12α+ cDC2s	0.786	0.583	0.640	0.796	0.291	0.736	0.756	0.945
MFI CD207+ cDC2s	0.445	0.669	0.257	0.276	0.678	0.953	0.716	0.889
MFI CD206+ cDC2s	0.157	0.003	0.231	0.020	0.054	0.135	0.100	0.472
% DCIR+ cDC1s	0.432	0.163	0.339	0.449	0.613	0.583	0.618	0.725
% Dectin-1+ cDC1s	0.605	0.752	0.709	0.798	0.420	0.897	0.706	0.632
% Clec-9α+ cDC1s	0.685	0.163	0.539	0.449	0.533	0.608	0.984	0.957
% Clec-12α+ cDC1s	1.000	0.795	1.000	0.795	0.586	0.040	0.116	0.083
% FcγRIIα+ cDC1s	1.000	0.180	1.000	0.180	0.590	0.619	0.695	0.695
% DEC-205+ cDC1s	1.000	0.795	1.000	0.795	0.586	0.040	0.116	0.083
% CD206+ cDC1s	1.000	0.089	1.000	0.089	0.013	0.040	0.116	0.083
MFI DCIR+ cDC1s	0.402	0.211	0.253	0.565	0.945	0.661	0.322	0.688
MFI Dectin-1+ cDC1s	0.097	0.187	0.007	0.056	0.261	0.879	0.706	0.263
MFI Clec-9α+ cDC1s	0.884	0.073	0.690	0.266	0.837	0.673	0.208	0.345
MFI Clec-12α+ cDC1s	1.000	0.180	1.000	0.180	0.716	0.619	0.886	0.918
MFI FcγRIIα+ cDC1s	1.000	0.937	1.000	0.937	0.013	0.040	0.116	0.083
MFI DEC-205+ cDC1s	1.000	0.795	1.000	0.795	0.977	0.508	0.695	0.967
MFI CD206+ cDC1s	1.000	1.000	1.000	1.000	0.343	0.040	0.116	0.018
% DCIR pDCs	0.951	0.757	0.442	0.784	0.678	0.953	0.716	0.889
% NKp44+ pDCs	0.497	0.044	0.321	0.173	0.494	0.751	0.475	0.814
% ILT7+ pDCs	0.001	0.019	0.079	0.111	0.228	0.620	0.756	0.932
% FcγRIIα+ pDCs	0.029	0.210	0.185	0.517	0.984	0.641	0.544	0.381
% FcεRIα+ pDCs	0.029	0.205	0.185	0.663	0.231	0.447	0.807	0.490
% BDCA2+ pDCs	0.290	0.074	0.185	0.375	0.412	0.840	0.846	0.810
MFI DCIR pDCs	0.147	0.601	0.948	0.981	0.271	0.483	0.183	0.197
MFI NKp44+ pDCs	0.693	0.320	0.767	0.262	0.172	0.020	0.424	0.444
MFI ILT7+ pDCs	0.138	0.456	0.259	0.883	0.305	0.658	0.997	0.749
MFI FcγRIIα+ pDCs	0.218	0.113	0.307	0.351	0.242	0.746	0.997	0.654
MFI FcεRIα+ pDCs	0.062	0.226	0.626	0.627	0.824	0.533	0.519	0.373
MFI BDCA2+ pDCs	0.138	0.265	0.259	0.699	0.500	0.831	0.909	0.751

<u>Supplementary table 7:</u> Correlation between CLR expression of DC subsets and their basal activation status in melanoma patients (Spearman correlation).

Spearman correlation (*r*, *P*-value)	Patient blood					
	% CD80+ cDC2s		% CD40+ cDC2s		% CD86+ cDC2s	
	r	*P*-value	*r*	*P*-value	*r*	*P*-value
% DCIR+ cDC2s	0.120	*0.646*	0.375	*0.139*	-0.174	*0.503*
% Dectin-1+ cDC2s	0.206	*0.426*	0.385	*0.128*	0.488	*0.049*
% DC-SIGN+ cDC2s	-0.076	*0.773*	-0.289	*0.259*	-0.176	*0.497*
% DEC-205+ cDC2s	0.321	*0.209*	0.414	*0.100*	-0.076	*0.773*
% Clec-12α+ cDC2s	-0.325	*0.203*	0.175	*0.501*	-0.433	*0.083*
% CD207+ cDC2s	-0.115	*0.660*	0.025	*0.928*	0.120	*0.646*
% CD206+ cDC2s	-0.145	*0.579*	0.127	*0.625*	0.196	*0.449*
MFI DCIR+ cDC2s	0.520	*0.032*	0.743	*0.001*	0.297	*0.247*
MFI Dectin-1+ cDC2s	0.172	*0.509*	0.137	*0.599*	-0.243	*0.347*
MFI DC-SIGN+ cDC2s	0.113	*0.666*	0.059	*0.824*	0.431	*0.085*
MFI DEC-205+ cDC2s	-0.083	*0.751*	0.250	*0.332*	0.015	*0.959*
MFI Clec-12α+ cDC2s	-0.078	*0.766*	-0.059	*0.824*	-0.382	*0.131*
MFI CD207+ cDC2s	-0.167	*0.521*	-0.110	*0.673*	-0.150	*0.566*
MFI CD206+ cDC2s	0.103	*0.694*	0.314	*0.220*	0.007	*0.981*

Spearman correlation (*r*, *P*-value)	Patient tumor infiltrate					
	% CD80+ cDC2s		% CD40+ cDC2s		% CD86+ cDC2s	
	r	*P*-value	*r*	*P*-value	*r*	*P*-value
% DCIR+ cDC2s	0.489	*0.093*	0.280	*0.353*	-0.336	*0.262*
% Dectin-1+ cDC2s	0.429	*0.146*	0.319	*0.289*	0.347	*0.246*
% DC-SIGN+ cDC2s	-0.187	*0.541*	0.005	*0.993*	0.151	*0.622*
% DEC-205+ cDC2s	0.473	*0.106*	0.027	*0.935*	-0.014	*0.964*
% Clec-12α+ cDC2s	0.137	*0.656*	0.088	*0.779*	0.063	*0.837*
% CD207+ cDC2s	-0.698	*0.010*	-0.401	*0.176*	0.127	*0.680*
% CD206+ cDC2s	-0.253	*0.404*	0.060	*0.849*	0.768	*0.002*
MFI DCIR+ cDC2s	0.665	*0.016*	0.231	*0.448*	-0.404	*0.171*
MFI Dectin-1+ cDC2s	0.044	*0.892*	-0.154	*0.617*	0.578	*0.039*
MFI DC-SIGN+ cDC2s	0.093	*0.765*	0.038	*0.906*	-0.402	*0.174*
MFI DEC-205+ cDC2s	0.198	*0.517*	-0.357	*0.232*	-0.003	*0.993*
MFI Clec-12α+ cDC2s	0.187	*0.541*	0.022	*0.949*	0.138	*0.654*
MFI CD207+ cDC2s	-0.319	*0.289*	-0.489	*0.093*	-0.124	*0.687*
MFI CD206+ cDC2s	-0.643	*0.021*	-0.346	*0.247*	0.275	*0.363*

Spearman correlation (*r*, *P*-value)	Patient blood					
	% CD80+ cDC1s		% CD40+ cDC1s		% CD86+ cDC1s	
	r	*P*-value	*r*	*P*-value	*r*	*P*-value
% DCIR+ cDC1s	0.551	*0.022*	0.098	*0.708*	0.382	*0.131*
% Dectin-1+ cDC1s	0.177	*0.496*	0.512	*0.038*	0.451	*0.071*
% Clec-9α+ cDC1s	0.330	*0.196*	0.132	*0.612*	0.049	*0.854*
MFI DCIR+ cDC1s	0.018	*0.945*	-0.471	*0.059*	0.002	*0.996*
MFI Dectin-1+ cDC1s	0.294	*0.253*	0.667	*0.004*	0.005	*0.989*
MFI Clec-9α+ cDC1s	0.145	*0.579*	-0.150	*0.566*	0.284	*0.268*

Spearman correlation (*r*, *P*-value)	Patient tumor infiltrate					
	% CD80+ cDC1s		% CD40+ cDC1s		% CD86+ cDC1s	
	r	*P*-value	*r*	*P*-value	*r*	*P*-value
% DCIR+ cDC1s	0.219	*0.544*	0.139	*0.707*	0.018	*0.973*
% Dectin-1+ cDC1s	-0.322	*0.364*	0.333	*0.349*	0.345	*0.331*
% Clec-9α+ cDC1s	-0.103	*0.776*	-0.018	*0.973*	0.248	*0.492*
MFI DCIR+ cDC1s	-0.517	*0.126*	0.200	*0.584*	0.030	*0.946*
MFI Dectin-1+ cDC1s	0.334	*0.345*	0.103	*0.785*	0.661	*0.044*
MFI Clec-9α+ cDC1s	-0.182	*0.614*	-0.345	*0.331*	0.467	*0.178*

Spearman correlation (*r*, *P*-value)	Patient blood					
	% CD80+ cDC1s		% CD40+ cDC1s		% CD86+ cDC1s	
	r	*P*-value	*r*	*P*-value	*r*	*P*-value
% DCIR pDCs	-0.015	*0.956*	0.547	*0.031*	0.468	*0.070*
% NKp44+ pDCs	0.313	*0.237*	-0.276	*0.299*	-0.503	*0.049*
% ILT7+ pDCs	0.090	*0.742*	0.444	*0.087*	-0.329	*0.213*
% FcγRIIα+ pDCs	-0.096	*0.725*	0.741	*0.001*	0.153	*0.571*
% FcεRIα+ pDCs	0.197	*0.465*	0.356	*0.176*	-0.365	*0.165*
% BDCA2+ pDCs	0.275	*0.303*	0.321	*0.226*	-0.174	*0.519*
MFI DCIR pDCs	0.242	*0.367*	0.224	*0.404*	-0.374	*0.155*
MFI NKp44+ pDCs	-0.215	*0.442*	0.311	*0.259*	0.221	*0.427*
MFI ILT7+ pDCs	-0.281	*0.292*	0.285	*0.283*	-0.100	*0.713*
MFI FcγRIIα+ pDCs	-0.006	*0.982*	0.503	*0.049*	0.065	*0.814*
MFI FcεRIα+ pDCs	0.358	*0.173*	0.132	*0.625*	-0.632	*0.010*
MFI BDCA2+ pDCs	-0.104	*0.700*	0.485	*0.059*	-0.168	*0.534*

Spearman correlation (*r*, *P*-value)	Patient tumor infiltrate					
	% CD80+ cDC1s		% CD40+ cDC1s		% CD86+ cDC1s	
	r	*P*-value	*r*	*P*-value	*r*	*P*-value
% DCIR pDCs	-0.423	*0.152*	-0.060	*0.849*	0.412	*0.163*
% NKp44+ pDCs	-0.301	*0.342*	-0.601	*0.043*	-0.238	*0.457*
% ILT7+ pDCs	-0.077	*0.807*	0.214	*0.482*	0.148	*0.630*
% FcγRIIα+ pDCs	-0.104	*0.737*	0.374	*0.209*	0.549	*0.055*
% FcεRIα+ pDCs	-0.022	*0.949*	-0.522	*0.071*	-0.126	*0.683*
% BDCA2+ pDCs	0.319	*0.289*	0.258	*0.394*	-0.104	*0.737*
MFI DCIR pDCs	0.099	*0.751*	0.000	*1.000*	-0.104	*0.737*
MFI NKp44+ pDCs	-0.336	*0.287*	-0.622	*0.035*	0.161	*0.619*
MFI ILT7+ pDCs	-0.165	*0.591*	0.247	*0.415*	0.363	*0.224*
MFI FcγRIIα+ pDCs	-0.302	*0.315*	-0.253	*0.404*	0.313	*0.297*
MFI FcεRIα+ pDCs	-0.610	*0.030*	-0.440	*0.135*	0.170	*0.579*
MFI BDCA2+ pDCs	-0.005	*0.993*	0.357	*0.232*	0.264	*0.383*

Supplementary table 8: Correlation between CLR expression of circulating DC subsets and DCs' cytokine production in melanoma patients (Spearman correlation).

Spearman correlation (*r*, *P*-value)	% IL-12p70/40+ cDC2s stim R848		% IL-12p70/40+ cDC2s stim mix		% TNFα+ cDC2s stim R848		% TNFα+ cDC2s stim mix	
	r	*P*-value	*r*	*P*-value	*r*	*P*-value	*r*	*P*-value
% DCIR+ cDC2s	0.425	*0.116*	0.235	*0.362*	0.646	*0.011*	0.510	*0.039*
% Dectin-1+ cDC2s	0.371	*0.173*	0.255	*0.322*	0.168	*0.549*	0.135	*0.605*
% DC-SIGN+ cDC2s	0.139	*0.621*	0.534	*0.029*	0.011	*0.974*	0.248	*0.337*
% DEC-205+ cDC2s	0.129	*0.648*	0.189	*0.467*	0.232	*0.404*	0.461	*0.065*
% Clec-12α+ cDC2s	0.502	*0.056*	0.679	*0.003*	0.718	*0.003*	0.522	*0.031*
% CD207+ cDC2s	-0.189	*0.498*	-0.211	*0.415*	-0.307	*0.265*	-0.083	*0.751*
% CD206+ cDC2s	0.111	*0.695*	0.324	*0.205*	0.064	*0.822*	0.257	*0.317*

Spearman correlation (*r*, *P*-value)	% IFNλ1+ cDC1s stim polyI :C		% IFNλ1+ cDC1s stim mix		% TNFα+ cDC1s stim polyI :C		% TNFα+ cDC1s stim mix	
	r	*P*-value	*r*	*P*-value	*r*	*P*-value	*r*	*P*-value
% DCIR+ cDC1s	0.257	*0.374*	-0.087	*0.749*	0.002	*1.000*	0.485	*0.059*
% Dectin-1+ cDC1s	-0.357	*0.191*	-0.186	*0.474*	-0.361	*0.187*	-0.179	*0.491*
% Clec-9α+ cDC1s	-0.213	*0.447*	0.134	*0.609*	-0.325	*0.237*	-0.056	*0.831*

Spearman correlation (*r*, *P*-value)	% IFNα+ pDCs stim R848		% IFNα+ pDCs stim CpGA		% IFNα+ pDCs stim mix		% TNFα+ pDCs stim R848		% TNFα+ pDCs stim CpGA		% TNFα+ pDCs stim mix	
	r	*P*-value	*r*	*P*-value	*r*	*P*-value	*r*	*P*-value	*r*	*P*-value	*r*	*P*-value
% DCIR pDCs	-0.314	*0.273*	-0.156	*0.611*	0.147	*0.586*	-0.437	*0.120*	0.016	*0.964*	-0.335	*0.204*
% NKp44+ pDCs	-0.235	*0.417*	0.039	*0.899*	-0.018	*0.952*	0.182	*0.532*	-0.148	*0.630*	0.497	*0.052*
% ILT7+ pDCs	0.029	*0.928*	-0.329	*0.273*	0.291	*0.273*	0.411	*0.146*	-0.335	*0.263*	0.338	*0.200*
% FcγRIIα+ pDCs	-0.196	*0.502*	-0.187	*0.541*	0.212	*0.430*	-0.231	*0.426*	-0.374	*0.209*	-0.132	*0.625*
% FcεRIα+ pDCs	-0.459	*0.101*	-0.326	*0.277*	-0.144	*0.594*	0.389	*0.170*	-0.505	*0.081*	0.241	*0.367*
% BDCA2+ pDCs	-0.336	*0.240*	-0.418	*0.155*	-0.015	*0.961*	-0.077	*0.797*	-0.467	*0.110*	-0.179	*0.505*
